# Absence of Additional Stretching‐Induced Electron Scattering in Highly Conductive Cross‐linked Nanocomposites with Negligible Tunneling Barrier Height and Width

**DOI:** 10.1002/advs.202409337

**Published:** 2024-10-28

**Authors:** C. Muhammed Ajmal, Juyeong Jeong, Seongsu Cheon, M. K. Majee, Heejun Yang, Seunghyun Baik

**Affiliations:** ^1^ Center for Nanotubes and Nanostructured Composites Sungkyunkwan University Suwon 16419 Republic of Korea; ^2^ Department of Physics Korea Advanced Institute of Science and Technology (KAIST) Daejeon 34141 Republic of Korea; ^3^ School of Mechanical Engineering Sungkyunkwan University Suwon 16419 Republic of Korea

**Keywords:** barrier height, barrier width, cross‐linked nanocomposites, resistance, tunneling

## Abstract

The intrinsic resistance of stretchable materials is dependent on strain, following Ohm's law. Here the invariable resistance of highly conductive cross‐linked nanocomposites over 53% strain is reported, where additional electron scattering is absent with stretching. The in situ generated uniformly dispersed small silver nanosatellite particles (diameter = 3.6 nm) realize a short tunneling barrier width of 4.1 nm in cross‐linked silicone rubber matrix. Furthermore, the barrier height can be precisely controlled by the gap state energy level modulation in silicone rubber using cross‐linkers. The negligible barrier height (0.01 eV) and short barrier width, achieved by the silver nanosatellite particles in cross‐linked silicone rubber, dramatically increase the electrical conductivity (51 710 S cm^−1^) by more than 4 orders of magnitude. The high conductance is also maintained over 53% strain. The quantum tunneling behavior is observed when the barrier height is increased, following the Simmons approximation theory. The transport becomes diffusive, following Ohm's law, when the barrier width is increased beyond 10.3 nm. This study provides a novel strain‐invariant resistance mechanism in highly conductive cross‐linked nanocomposites.

## Graphical abstract

The invariable resistance of highly conductive cross‐linked nanocomposites over 53% strain is reported, where additional electron scattering is absent with stretching. The negligible barrier height (0.01 eV) and short barrier width, achieved by the silver nanosatellite particles in silicone rubber, dramatically increase the conductivity (51 710 S cm^−1^) by more than 4 orders of magnitude.

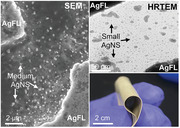

## 1 Introduction

Stretchable conductive nanocomposites have received considerable attention as building blocks for wearable electronics, skin electronics, sensors, and soft robotics.^[^ ^1^, ^2^, ^3^, ^4^ ^]^ They are typically made of conductive nanoparticles embedded in a polymer matrix.^[^ ^5^, ^6^, ^7^ ^]^ The electrical transport through these nanocomposites is diffusive, following Ohm's law. The intrinsic resistance of stretchable conductive nanocomposites is dependent on strain. However, a constant resistance with stretching is desirable for electrode applications.^[^ ^3^, ^8^ ^]^ Various approaches have been investigated to realize strain‐insensitive resistance. Among these are external geometrical modifications such as kirigami‐inspired structures,^[^ ^9^, ^10^ ^]^ serpentine designs,^[^ ^11^ ^]^ and surface wrinkling/buckling techniques.^[^ ^12^ ^]^ Nevertheless, the intrinsic electrical transport of these nanocomposites is still diffusive, and thus, their intrinsic resistance increases by stretching. The fluidity of liquid metals can also provide strain‐insensitive resistance for nanocomposites although potential leakage and oxidation impede their applications.^[^ ^13^ ^,^ ^14^ ^]^

A high conductivity is also a necessity for stretchable conductive nanocomposites. The construction of an efficient percolation network has been studied‐based on the direct physical contact of conductive fillers such as carbon nanomaterials,^[^ ^15^ ^]^ metal nanoparticles/wires,^[^ ^16^, ^17^ ^]^ and their mixtures.^[^ ^18^, ^19^, ^20^ ^]^ Nevertheless, a significant portion of conductive fillers still remains isolated in the matrix polymer, hindering their contribution to electron transport.^[^ ^5^, ^6^, ^21^ ^]^ The electron tunneling can occur if the insulating polymer between noncontacted particles is sufficiently thin (e.g., <10 nm).^[^ ^5^, ^6^, ^7^, ^21^, ^22^, ^23^, ^24^ ^]^ The electron tunneling current is governed by the potential energy barrier height and width (i.e., interparticle distance), and it rapidly decreases with increasing tunneling width following the Simmon's approximation theory.^[^ ^22^ ^]^

The quantum tunneling in conductive nanocomposites is not yet well understood, due to the random nature of particle size and dispersion.^[^ ^5^, ^7^, ^21^, ^23^, ^24^ ^]^ We recently reported uniformly dispersed small silver nanosatellite (AgNS) particles (diameter <5 nm) generated by the free radical and reactive oxygen species‐mediated in situ etching and reduction reaction of Ag flakes (AgFLs) using tetrahydrofuran (THF) peroxide.^[^ ^5^, ^6^ ^]^ The interparticle distance was also small (<5 nm).^[^ ^5^, ^6^ ^]^ The AgNS particles dispersed in an uncrosslinked silicon rubber (SR) matrix provided an invariant resistance of up to 30% strain.^[^ ^5^ ^]^ However, the transport mechanism was not clarified in the work, which deviated from the Ohm's law and Simmons approximation theory for quantum tunneling.^[^ ^5^, ^6^ ^]^ Furthermore, the nanocomposite exhibited very low electrical conductivity (*σ* = ≈12 S cm^−1^) and mechanical strength (0.02 MPa) due to the large voids and viscoelastic putty‐like nature of the uncrosslinked SR.^[^ ^5^ ^]^

Here we report the invariable resistance of highly conductive cross‐linked nanocomposites over 53% strain, where additional electron scattering is not made by stretching. The in situ generated uniformly dispersed small AgNS particles (diameter = 3.6 nm) realize a short barrier width of 4.1 nm in a cross‐linked SR matrix. Furthermore, the barrier height (0.01–0.14 eV) is precisely controlled by the SR gap state energy level modulation using cross‐linkers (0–10 wt.%), which is accurately analyzed by Kelvin probe force microscopy (KPFM). The cross‐linking forms strong C─C covalent bonds, removing voids and significantly increasing mechanical strength. The negligible barrier height (0.01 eV) and short barrier width (4.1 nm) dramatically increase the *σ* (51 710 S cm^−1^) of the cross‐linked nanocomposite (Ag = 40 vol.%) by more than 4 orders of magnitude, compared with an uncrosslinked nanocomposite with a high barrier height (0.14 eV). The high conductance does not change over 53% strain. The negative magnetoresistance analysis, successfully fitted with the Hikami‐Larkin‐Nagaoka equation, further supports the unique strain‐invariant resistance behavior. The current exponentially decreases with stretching when the barrier height is increased, following the Simmons approximation theory for quantum tunneling. When the barrier width between AgNS particles is increased beyond 10.3 nm, the transport becomes diffusive. The resistance is then dominated by the scatterings in the channel and increases by the longer channel length and smaller cross‐section, following the conventional Ohm's law. This study provides a counter‐intuitive strain‐independent resistance mechanism in highly conductive cross‐linked nanocomposites.

## 2 Results and Discussion

### 2.1 The Barrier Width Modulation by AgNS Particles

**Figure 1:**
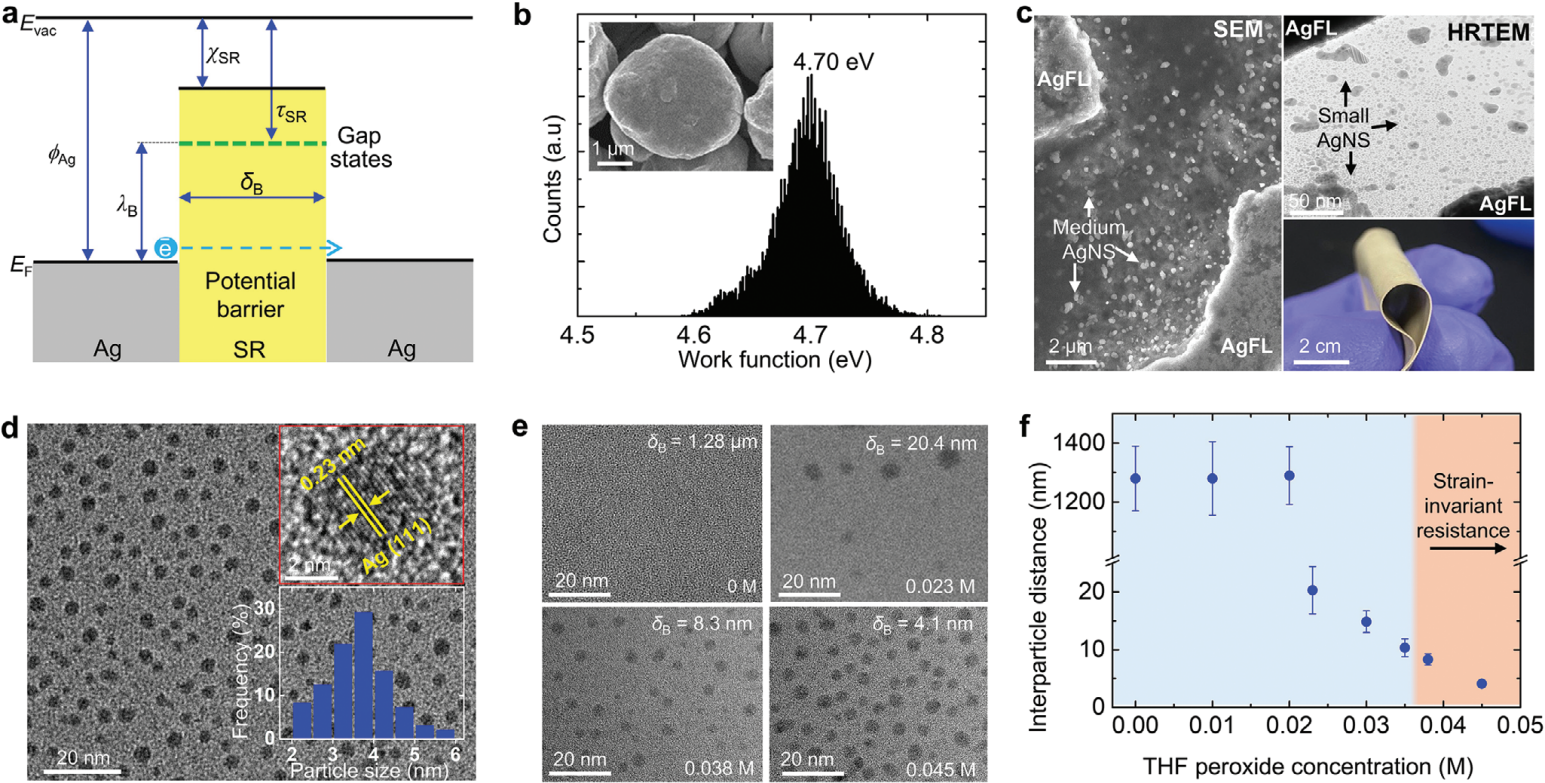
The barrier width modulation by AgNS particles. a) Ag‐SR‐Ag tunneling junction diagram. b) The work function distribution of AgFLs. The SEM image of AgFLs is provided in the inset. c) The SEM and HRTEM images of the cross‐linked AgFL‐AgNS‐SR_DDTP_7wt.%_ nanocomposite showing medium and small AgNS particles (AgFL‐AgNS = 40 vol.%). An optical image of the nanocomposite is also provided. d) The HRTEM image of small AgNS particles. The lattice resolved HRTEM image of a single AgNS particle and particle size distribution are provided as insets. e,f) The HRTEM images and average barrier width between small AgNS particles (*δ*_B_) are shown as a function of the THF peroxide concentration (AgFL‐AgNS‐SR_DDTP_7wt.%_, AgFL‐AgNS = 40 vol.%).

Quantum tunneling occurs in a metal‐insulator‐metal junction when the thickness of the insulator (i.e., barrier width (*δ*_B_)) is sufficiently small (**Figure** **1**a).^[^ ^5^, ^22^, ^25^ ^]^ The Fermi levels are aligned when the materials are in contact at equilibrium. The tunneling current density (*J*) is described by the Simmons approximation theory when the applied voltage across the electrodes (*V*) is sufficiently low.^[^ ^5^, ^22^, ^26^ ^]^

(1) $$J=\frac{3{\left(2m_{e}\lambda_{B}\right)}^{\frac{1}{2}}}{2\delta_{B}}\;{\left(\frac{e}{h}\right)}^{2}\;V\exp\left(-\frac{4\pi \delta_{B}}{h}\;{\left(2m_{e}\lambda_{B}\right)}^{\frac{1}{2}}\right)$$

where *m*_e_ is the effective mass of an electron, *h* is Planck's constant, and *e* is the elementary charge.^[^ ^22^ ^]^ For ideal insulating polymers, the barrier height (*λ*_B_) is determined by the difference between the work function of Ag (*ϕ*_Ag_) and electron affinity of SR (*χ*_SR_).^[^ ^22^, ^27^ ^]^ However, in reality, polymers have numerous energy states within the bandgap owing to structural defects, chemical impurities, adsorbed molecules, and cross‐linking agents.^[^ ^28^, ^29^, ^30^ ^]^ It is assumed that the gap states can form a conduction path within the bandgap if *δ*_B_ is sufficiently small (<10 nm). In such case, *λ*_B_ is determined by the energy level of the gap states (*τ*_SR_), resulting in *λ*_B_ = *ϕ*_Ag –_ *τ*_SR_ (Figure 1a).^[^ ^22^, ^29^ ^]^

The *ϕ*_Ag_ is measured by KPFM using a gold‐coated silicon tip (Figure 1b). The AgFLs are deposited on a silicon wafer, and the measurement is calibrated using highly ordered pyrolytic graphite (HOPG, work function = 4.6 eV).^[^ ^5^, ^31^ ^]^ The topography and contact potential difference (*V*_CPD_) are provided in Figure S1 (Supporting Information). The average *ϕ*_Ag_ is found to be 4.70 eV. A scanning electron microscopy (SEM) image of AgFLs is also shown (Figure 1b, inset).

The tunneling current decreases rapidly with increasing *δ*_B_ as discussed in Equation (1).^[^ ^5^ ^]^ Therefore, it is important to achieve a small *δ*_B_ to induce high conductivity of nanocomposites. The AgNS particles are generated using THF peroxide following a previously published protocol.^[^ ^5^, ^6^ ^]^ The concentration of THF peroxide is precisely controlled by reacting THF with atmospheric oxygen through the air bubbling process.^[^ ^5^, ^32^ ^]^ The THF peroxide concentration increases as the air bubbling time increases, reaching 0.045 m after 72 h (Figure S2, Supporting Information). The synthesis schematic of the AgFL‐AgNS‐SR_DDTP_ nanocomposite is shown in Figure S3 (Supporting Information), and a detailed procedure is provided in the Experimental Section. Briefly, AgFLs, THF peroxide, SR, and cross‐linking agent (2,5‐dimethyl‐2,5‐di(tert‐butylperoxy)hexane (DDTP)) are mixed and reacted for 45 min. The mixture is then drop‐casted and dried overnight followed by hot‐pressing (170 °C, 3 MPa, 10 min) and curing (200 °C, 4 h). The detailed Ag‐THF peroxide reaction mechanism was provided previously.^[^ ^6^ ^]^ Briefly, 2‐hydroxytetrahydrofuran and oxygen molecules are formed as the reaction byproducts.^[^ ^6^ ^]^ The byproducts are removed during the hot‐pressing and curing process since the boiling point of 2‐hydroxytetrahydrofuran is lower than 165 °C. The SEM and high‐resolution transmission electron microscopy (HRTEM) images of the cross‐linked AgFL‐AgNS‐SR_DDTP_7wt.%_ nanocomposite (AgFL‐AgNS = 40 vol.%, relative concentration of DDTP in SR = 7 wt.%) show medium and small AgNS particles generated by the THF peroxide reaction (Figure 1c). The medium AgNS particles are uniformly distributed between AgFLs, and the average particle size is found to be 139 nm (Figure S4, Supporting Information)). A magnified HRTEM image shows uniformly distributed small AgNS particles, around the medium AgNS particles, which are crucial for the efficient electron tunneling (Figure 1d). A lattice resolved HRTEM image of a small AgNS particle confirms the interplanar distance corresponding to the Ag (111) plane, and the average size is 3.6 nm (Figure 1d, inset). The generation of AgNS particles does not change the total Ag concentration because they come from the vigorous in situ etching and reduction of AgFLs.^[^ ^5^, ^6^ ^]^ The rough surface of AgFLs embedded in the AgFL‐AgNS‐SR_DDTP_7wt.%_ nanocomposite is shown in Figure S5 (Supporting Information). Note that the cross‐linked AgFL‐AgNS‐SR_DDTP_7wt.%_ nanocomposite does not have any void, unlike the previously reported viscoelastic putty‐like nanocomposites.^[^ ^5^, ^6^ ^]^ The organic peroxide (DDTP) mediated cross‐linking of SR chains increases the *σ* and mechanical strength by orders of magnitude as will be discussed shortly.

The concentration of THF peroxide plays an important role in the degree of AgFL etching and AgNS particle generation.^[^ ^5^ ^]^ Figure 1e compares HRTEM images and corresponding interparticle distance (i.e., *δ*_B_) of the AgFL‐AgNS‐SR_DDTP_7wt.%_ nanocomposite as a function of the THF peroxide concentration (0–0.045 m). The average *δ*_B_ between the surfaces of adjacent AgNS particles is measured from the HRTEM images. There is no AgNS particle generation when the nanocomposite is synthesized using THF with a butylated hydroxytoluene (BHT) peroxidation inhibitor (i.e., THF peroxide = 0 m).^[^ ^5^, ^6^ ^]^ The corresponding distance between AgFLs is *δ*_B_ = 1.28 µm. The AgNS particles are observed when the THF peroxide concentration increases beyond 0.02m (Figures 1e and S6, Supporting Information). The average size of small AgNS particles generally decreases, although there is somewhat variation, as the THF peroxide concentration increases (Figure S7, Supporting Information). This is due to the vigorous etching of AgFLs at a higher THF concentration. The *δ*_B_ also decreases as the THF peroxide concentration increases, reaching *δ*_B_ = 4.1 nm at THF peroxide = 0.045 m (Figure 1f). The smaller particle size and *δ*_B_ are favorable to increase electrical conductivity. The independent control of particle size and *δ*_B_ needs to be investigated further in the future. The strain‐invariant resistance could be observed when *δ*_B_ <10.5 nm as will be discussed shortly.

The number density of small AgNS particles is shown as a function of the THF peroxide concentration (Figure S8, Supporting Information). The number density increases as the THF peroxide concentration increases, reaching 1.06 × 10^4^ particles µm^−^^2^ at THF peroxide = 0.045 m. The volume fractions of small and medium AgNS particles in the SR matrix could be roughly estimated using the average particle size (*s*) and interparticle distance (*d_0_*).^[^ ^5^ ^]^ The total volume of all Ag particles in the nanocomposite after the THF peroxide reaction (0.045 m) is 40 vol.%, which is the sum of AgFLs, medium AgNS particles, and small AgNS particles. It is difficult to precisely estimate the volume fraction of AgNS particles since they are generated by the in situ etching and reduction reaction of AgFLs inside the nanocomposite. As a rough approximation, the small spherical AgNS particles (*s* = 3.6 nm, *d_0_* = 4.1 nm) are assumed to be arranged in a simple cubic structure in the polymer matrix as shown in Figure S9 (Supporting Information).^[^ ^5^ ^]^ This results in 3.21 vol.% for small AgNS particles in the entire nanocomposite. Similarly, the volume fraction of medium AgNS particles (*s* = 139 nm, *d_0_* = 208 nm) is estimated to be 2.02 vol.% in the entire nanocomposite. Then, the volume fraction of AgFLs is calculated to be 34.77 vol.%.

### 2.2 The Barrier Height Modulation by Cross‐linking of SR

**Figure 2:**
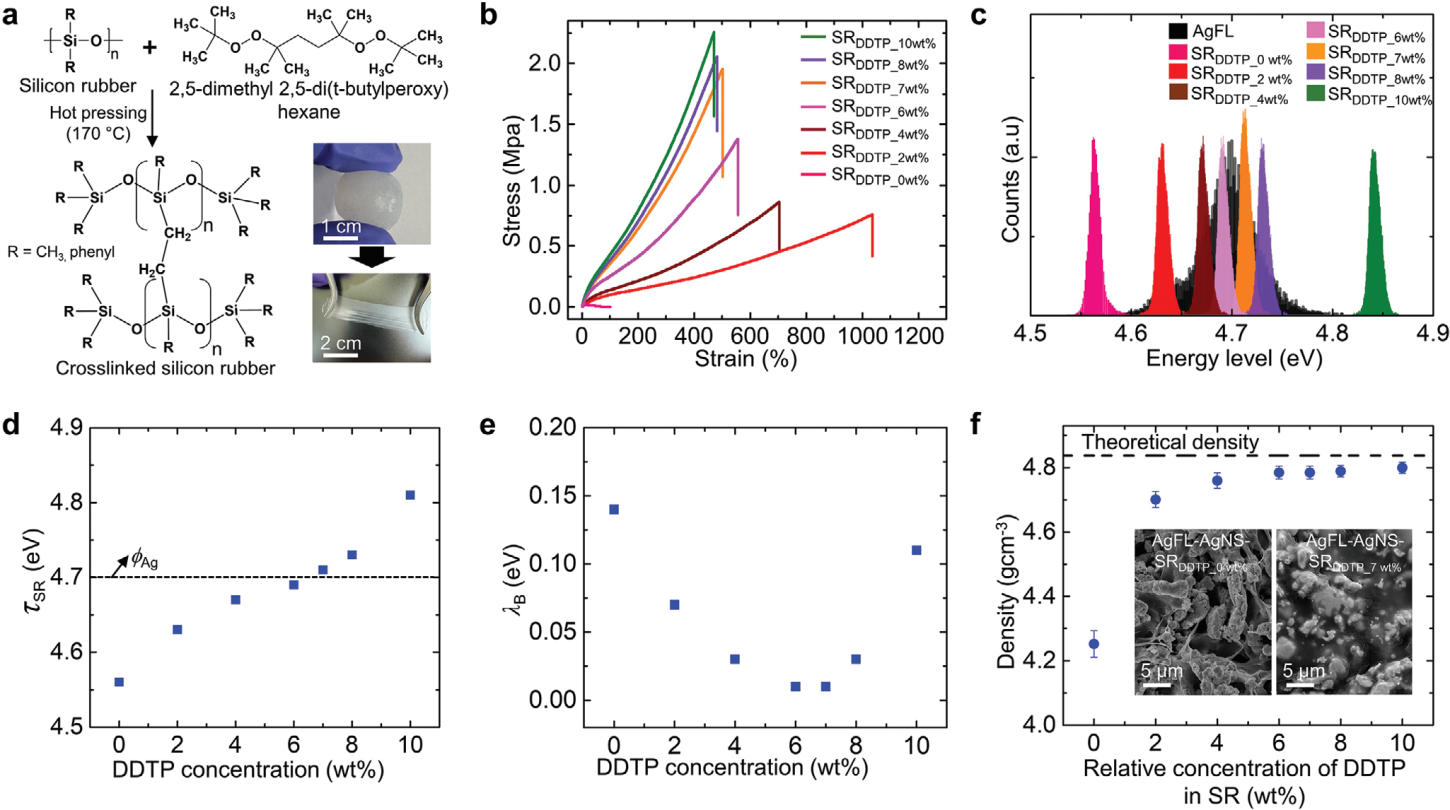
The barrier height modulation by cross‐linking of SR. a) Schematic of the cross‐linking process. Optical images of SR before and after cross‐linking are also provided. b) The tensile stress‐strain characteristics of the SR with different DDTP concentrations (0‐10 wt.%). c) The KPFM measurements: *Φ*_Ag_ and *τ*_SR_ (SR_DDTP_0wt.%_‐SR_DDTP_10wt.%_). d,e) The mean *τ*_SR_ and corresponding *λ*_B_ (SR_DDTP_0wt.%_‐SR_DDTP_10 wt.%_). f) The density of the AgFL‐AgNS‐SR_DDTP_ nanocomposite (AgFL‐AgNS = 40 vol.%) is shown as a function of the relative concentration of DDTP in SR (SR_DDTP_0wt.%_‐SR_DDTP_10wt.%_). The cross‐sectional SEM images are provided as insets (SR_DDTP_0wt.%_ and SR_DDTP_7wt.%_).

The barrier height (*λ*_B_) is a critical parameter for electron tunneling, in addition to *δ*_B_, as discussed in Equation (1). The *λ*_B_ can be precisely controlled by cross‐linking SR using an organic peroxide‐based cross‐linker DDTP^[^ ^33^ ^]^ (**Figure** **2**a). The DDTP is selected since the free‐radical initiated cross‐linking mechanism does not affect the inherent stability of the polymer.^[^ ^34^ ^]^ A detailed synthesis procedure is provided in the Experimental Section. Briefly, the pristine SR is mixed with DDTP followed by hot‐pressing (170 °C, 3 MPa, 10 min). The resulting product is further cured at 200 °C for 4 h to obtain the cross‐linked SR. The pristine SR has a viscoelastic putty‐like nature and makes a permanent shape change upon stretching.^[^ ^5^ ^]^ However, it becomes highly stretchable and elastic after cross‐linking (Figure 2a, inset). The cross‐linking density is measured by the equilibrium swelling method as a function of the DDTP concentration in SR (Figure S10, Supporting Information).^[^ ^35^, ^36^, ^37^ ^]^ The cross‐linking density is significantly increased from 3.67 × 10^−6^ to 1.64 × 10^−4^ mol cm^−3^ as the DDTP concentration increases from 0 to 10 wt.%, owing to new strong C─C covalent bond formation.

The mechanical stress‐strain characteristics are investigated as a function of the DDTP concentration in SR (SR_DDTP_0wt.%_‐SR_DDTP_10wt.%_) as shown in Figure 2b. The tensile strength of the pristine SR (SR_DDTP_0wt.%_) is only 0.014 MPa with a rupture strain of 102%. The cross‐linking significantly increases both tensile strength and rupture strain. The C─C covalent bonds formed by cross‐linking increase the tensile strength by more than 2 orders of magnitude for SR_DDTP_7wt.%_‐SR_DDTP_10wt.%_.

The KPFM measures *V*_CPD_ between the tip and specimen: −e*V*_CPD_ = * ϕ*_tip –_ *ξ*_specimen_. The *ϕ*_tip_ is the work function of the tip, and *ξ*_specimen_ is the difference between the vacuum level and highest electron‐occupied energy level of the specimen.^[^ ^38^, ^39^ ^]^ For metals, *ξ*_specimen_ is the work function of the specimen (e.g., *ϕ*_Ag_).^[^ ^38^, ^39^, ^40^ ^]^ For ideal insulating polymers, the valence band is completely filled with electrons, and the conduction band is completely empty with a large and perfect energy bandgap. However, in reality, polymers have numerous energy states within the bandgap as discussed in Figure 1a. In such case, *ξ*_specimen_ would be the difference between the vacuum level and the energy level of the gap states (i.e., *τ*_SR_).

Figure 2c shows *τ*_SR_ as a function of the DDTP concentration in SR (SR_DDTP_0wt.%_‐SR_DDTP_10wt.%_). Thin SR_DDTP_wt.%_ films are transferred on a silicon wafer for the KPFM measurement. Note that the SR_DDTP_wt.%_ films do not have embedded Ag particles. The *ϕ*_Ag_ is also shown for comparison. The *τ*_SR_ of pristine SR (i.e., SR_DDTP_0wt.%_) is 4.56 eV. Interestingly, *τ*_SR_ gradually increases by cross‐linking as the DDTP concentration increases. This is attributed to the creation of additional energy levels within the bandgap (gap states) by cross‐linking.^[^ ^41^ ^]^ The cross‐linking results in structural defects including new bond formation, byproducts, and changes in crystallinity and morphology.^[^ ^41^, ^42^ ^]^ The average *ϕ*_Ag_ and *τ*_SR_ are compared in Figure 2d. Figure 2e shows *λ*_B_ = *ϕ*_Ag –_ *τ*_SR_ as a function of the DDTP concentration in SR. The overlap between *ϕ*_Ag_ and *τ*_SR_, with cross‐linking, results in negligible *λ*_B_ values (≈0.01 eV) for SR_DDTP_6wt.%_ and SR_DDTP_7wt.%_. However, the *λ*_B_ is increased as the DDTP concentration increases beyond 7 wt.%. The *λ*_B_ values are 0.03 and 0.11 eV for SR_DDTP_8wt.%_ and SR_DDTP_10wt.%_, respectively.

Figure 2f shows the experimentally measured density of the AgFL‐AgNS‐SR_DDTP_ nanocomposites as a function of the relative DDTP concentration in SR. The total concentration of AgFL‐AgNS particles is fixed at 40 vol.% in all the nanocomposites. The experimentally measured density of the AgFL‐AgNS‐SR_DDTP_0wt.%_ nanocomposite is significantly smaller than the theoretical density calculated by the rule of mixture. Without cross‐linking, the linear polymer chains of SR_DDTP_0wt.%_ are connected by the relatively weak hydrogen bonding only.^[^ ^5^ ^]^ This results in large voids as shown in the cross‐sectional SEM images of the AgFL‐AgNS‐SR_DDTP_0wt.%_ nanocomposite (Figure 2f, inset; Figure S11, Supporting Information). The weak hydrogen bonding and voids substantially compromise mechanical strength and electrical conductivity.^[^ ^5^, ^6^ ^]^ The experimentally measured density increases as the relative DDTP concentration increases. A close agreement (<1.1%) between the experimental and theoretical densities is observed when the DDTP concentration is >6 wt.%. Any void is not observed in the cross‐linked AgFL‐AgNS‐SR_DDTP_7wt.%_ nanocomposite. The newly formed strong C─C covalent bonds by cross‐linking remove voids and increase mechanical strength and electrical conductivity, as will be discussed shortly.

**Figure 3:**
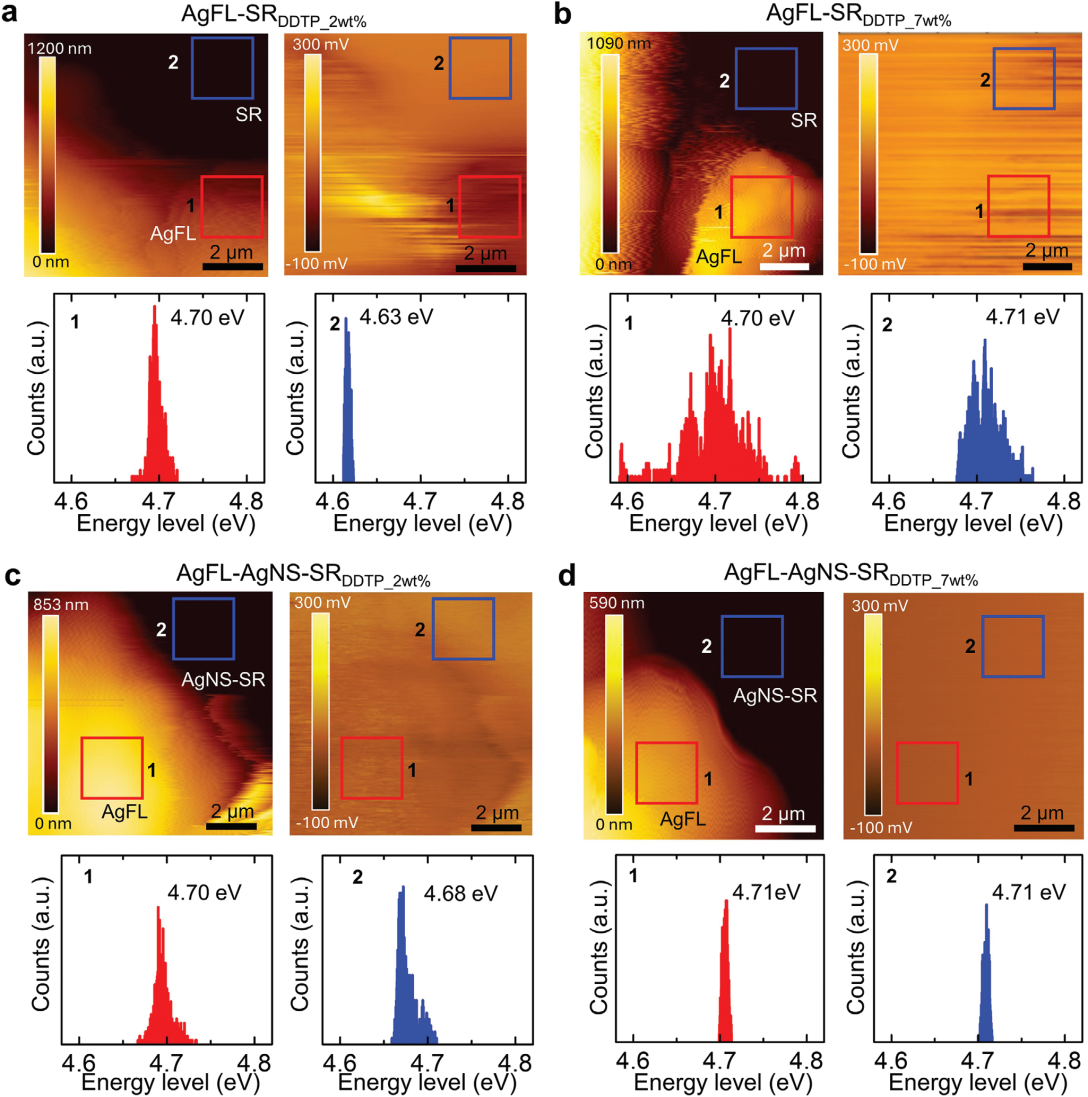
The KPFM topography and corresponding contact potential difference of the AgFL‐SR_DDTP_ and AgFL‐AgNS‐SR_DDTP_ nanocomposites (Ag = 40 vol.%). A gold‐coated silicon tip is used, and the contact potential difference distributions in the marked square regions (1–2) are also provided. a) AgFL‐SR_DDTP_2wt.%_. b) AgFL‐SR_DDTP_7wt.%_. c) AgFL‐AgNS‐SR_DDTP_2wt.%_. d) AgFL‐AgNS‐SR_DDTP_7wt.%_.

**Figure** **3** shows the KPFM area mapping of the AgFL‐SR_DDTP_ and AgFL‐AgNS‐SR_DDTP_ nanocomposites. The AgFL‐SR_DDTP_ nanocomposite does not have AgNS particles since it is synthesized using the THF with BHT inhibitor (THF peroxide = 0 m). The total Ag concentration is identical for both nanocomposites (Ag = 40 vol.%). The SR_DDTP_2wt.%_ and SR_DDTP_7wt.%_ are selected as matrix polymers to represent non‐negligible (0.07 eV) and negligible (0.01 eV) barrier heights, respectively (Figure 2e). The SR_DDTP_0wt.%_ and SR_DDTP_10wt.%_ are excluded for the matrix polymers of non‐negligible barrier height nanocomposites although they provide higher *λ*_B_ values (0.11 and 0.14 eV). There are large voids in the SR_DDTP_0wt.%_ matrix nanocomposite, resulting in poor electrical and mechanical properties. The SR_DDTP_10wt.%_ matrix nanocomposite suffers from low stretchability (≈8%). The nanocomposites are frozen using liquid nitrogen, cut, and polished for the cross‐sectional KPFM measurements.

Figure 3a shows the topography and *V*_CPD_ of the AgFL‐SR_DDTP_2wt.%_ nanocomposite. The corresponding energy values in the marked square regions 1 and 2 are also shown. The average *ϕ*_Ag_ on an AgFL (region 1) is 4.70 eV, and the average *τ*_SR_ in the matrix polymer region 2 is 4.63 eV. These precisely agree with the values separately measured on the pure AgFLs and pure SR_DDTP_2wt.%_ film (Figure 2d), resulting in *λ*_B_ = 0.07 eV. The topography and *V*_CPD_ of the AgFL‐SR_DDTP_7wt.%_ nanocomposite are shown in Figure 3b. The average *ϕ*_Ag_ on an AgFL (region 1) is 4.70 eV again although the standard deviation is a bit larger. The average *τ*_SR_ in the matrix polymer region 2 is 4.71 eV, which is consistent with the value separately measured on the pure SR_DDTP_7wt.%_ film (Figure 2d). It is worth noting that the *σ* of the AgFL‐SR_DDTP_7wt.%_ nanocomposite is small due to the large *δ*_B_, despite the negligible *λ*_B_, as will be discussed later.

In the case of the AgFL‐AgNS‐SR_DDTP_2wt.%_ nanocomposite, the average energy on region 2 is increased to 4.68 eV (Figure 3c). This is because small AgNS particles (3.6 nm) are uniformly distributed with an interparticle distance of 4.1 nm in SR_DDTP_2wt.%_. The KPFM tip provides an average value due to the spatial resolution limitation of the measurement. A very uniform *V*_CPD_ map is observed for the AgFL‐AgNS‐SR_DDTP_7wt.%_ nanocomposite (Figure 3d). The *ϕ*_Ag_ and *τ*_SR_ of SR_DDTP_7wt.%_ are very similar, resulting in a negligible *λ*_B_ (≈0 eV). The negligible *λ*_B_, together with the small *δ*_B_ (4.1 nm), leads to the extraordinarily high *σ* of the AgFL‐AgNS‐SR_DDTP_7wt.%_ nanocomposite as will be discussed shortly.

### 2.3 Electrical Transport of the AgFL‐AgNS‐SR_DDTP_7wt.%_ Nanocomposite

**Figure 4:**
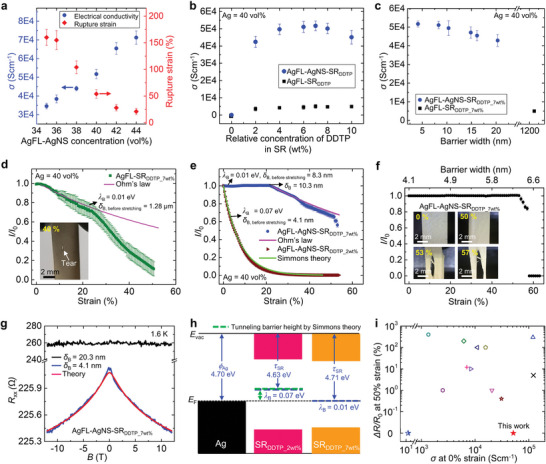
Electrical transport of the AgFL‐AgNS‐SR_DDTP_7wt.%_ nanocomposite. a) The *σ* and rupture strain of the AgFL‐AgNS‐SR_DDTP_7wt.%_ nanocomposite as a function of the AgFL‐AgNS concentration. b) The *σ* of the AgFL‐SR_DDTP_ and AgFL‐AgNS‐SR_DDTP_ nanocomposites (Ag = 40 vol.%) as a function of the relative DDTP concentration in SR (SR_DDTP_0wt.% –_ SR_DDTP_10wt.%_). c) The *σ* of the AgFL‐SR_DDTP_7wt.%_ and AgFL‐AgNS‐SR_DDTP_7wt.%_ nanocomposites (Ag = 40 vol.%) as a function of the initial barrier width. d) The normalized current of the AgFL‐SR_DDTP_7wt.%_ (THF peroxide = 0 m) nanocomposite as a function of tensile strain (applied voltage = 10 mV, crosshead speed = 1 mm min^−1^). The theoretical prediction by Ohm's law, assuming constant resistivity, is also shown. Inset shows the optical microscopic image of the nanocomposite at 40% strain. e) The normalized current of the AgFL‐AgNS‐SR_DDTP_7wt.%_ (THF peroxide = 0.038 m) and AgFL‐AgNS‐SR_DDTP_2wt.%_ (THF peroxide = 0.045 m) as a function of tensile strain (applied voltage = 10 mV, crosshead speed = 1 mm min^−1^). The theoretical predictions by the Simmons approximation model (AgFL‐AgNS‐SR_DDTP_2wt.%_) and Ohm's law (AgFL‐AgNS‐SR_DDTP_7wt.%,_ strain >10.3%) are also shown. f) The normalized current of the AgFL‐AgNS‐SR_DDTP_7wt.%_ nanocomposite (AgFL‐AgNS = 40 vol.%, THF peroxide = 0.045 m) as a function of tensile strain (applied voltage = 10 mV, crosshead speed = 1 mm min^−1^). Inset shows the optical microscopic images of the nanocomposite. g) Magnetoresistance of the AgFL‐AgNS‐SR_DDTP_7wt.%_ nanocomposites (AgFL‐AgNS = 40 vol.%) with *δ*_B_ = 4.1 nm (THF peroxide = 0.045 m) and 20.3 nm (THF peroxide = 0.023 m). The negative magnetoresistance is fitted by the Hikami‐Larkin‐Nagaoka equation. h) The energy band alignment diagram of the Ag, SR_DDTP_2wt.%_, and SR_DDTP_7wt.%_. The tunneling barrier heights obtained by the KPFM measurement (blue) and Simmons approximation fitting (green dash line) are compared. i) The *σ* at 0% strain and normalized resistance change at 50% strain of the AgFL‐AgNS‐SR_DDTP_7wt.%_ (AgFL‐AgNS = 40 vol.%, THF peroxide = 0.045 m) nanocomposite (solid red star) are compared with those of stretchable conductive nanocomposites in literature (open symbols). The AgFL‐AgNS‐SR nanocomposite from our previous study, which was not crosslinked with a rupture strain of ≈30%, is also added for comparison (half‐filled blue star).^[^ ^5^ ^]^ The open symbols are dark cyan circle,^[^ ^46^ ^]^ purple hexagon,^[^ ^48^ ^]^ olive diamond,^[^ ^8^ ^]^ left pointing triangle,^[^ ^50^ ^]^ dark yellow pentagon,^[^ ^17^ ^]^ violet right pointing triangle,^[^ ^49^ ^]^ pink cross,^[^ ^45^ ^]^ magenta down pointing triangle,^[^ ^13^ ^]^ blue up pointing triangle,^[^ ^47^ ^]^ wine star,^[^ ^20^ ^]^ and black cross.^[^ ^16^ ^]^

**Figure** **4**a shows the *σ* and rupture strain of the AgFL‐AgNS‐SR_DDTP_7wt.%_ nanocomposite as a function of the total Ag (i.e., AgFL‐AgNS) concentration. The *σ* of the rectangular‐shaped specimen (length = 20 mm, width = 5 mm, thickness = 0.1–0.2 mm) is measured by the four‐point method (see Experimental Section for details).^[^ ^18^, ^19^ ^]^ The *σ* increases as the AgFL‐AgNS concentration increases, reaching 71 245 S cm^−1^ at AgFL‐AgNS = 44 vol.%. However, the rupture strain decreases with increasing Ag concentration. Therefore, the AgFL‐AgNS concentration can be optimized at 40 vol.% considering both conductivity and stretchability (*σ* = 51 710 S cm^−1^ and rupture strain = 54%). It is important to uniformly disperse fine conductive nanoparticles within the tunneling cutoff distance to obtain high conductivity. However, it is very challenging to achieve uniform dispersion with a small *δ*_B_ (≈4 nm) by directly mixing small Ag nanoparticles (≈4 nm) with matrix polymer due to the particle aggregation. This highlights the unique advantage of the in situ generated AgNS particles.

The effect of *λ*_B_ on the *σ* of the AgFL‐SR_DDTP_ and AgFL‐AgNS‐SR_DDTP_ nanocomposites (total Ag concentration = 40 vol.%) is investigated as a function of the relative DDTP concentration in SR (SR_DDTP_0wt.%_ – SR_DDTP_10wt.%_) as shown in Figure 4b. The *σ* of the AgFL‐SR_DDTP_0wt.%_ nanocomposite is only 0.12 S cm^−1^ without any cross‐linking. Note that there are large voids in the uncrosslinked SR matrix nanocomposite.^[^ ^5^ ^]^ The *σ* increases to 3541 S cm^−1^ for the AgFL‐SR_DDTP_2wt.%_ nanocomposite. A small addition of DDTP cross‐linker effectively decreases the void concentration. However, there is no further significant increase in *σ* even with higher DDTP concentrations (AgFL‐SR_DDTP_4wt.%_‐AgFL‐SR_DDTP_8wt.%_) although the *λ*_B_ becomes negligible (0.01–0.03 eV). This is due to the absence of AgNS particles and large *δ*_B_ (≈1.28 µm) in the AgFL‐SR_DDTP_ nanocomposites. The gap states exhibit poor mobility and cannot make efficient electron transport over such a long distance. In contrast, the *σ* of the AgFL‐AgNS‐SR_DDTP_ nanocomposite significantly increases from 1.67S cm to 42 420 S cm^−1^ as the relative DDTP concentration in SR increases from 0 to 2 wt.% (Figure 4b). A number of factors contribute together to the large increase in *σ*. First, the void concentration is decreased by cross‐linking. Second, the *λ*_B_ is decreased from 0.14 to 0.07 eV. Finally, the *δ*_B_ is only 4.1 nm due to the presence of AgNS particles (THF peroxide = 0.045 m). The *σ* further increases to 51 710 S cm^−1^ as the *λ*_B_ is decreased to 0.01 eV for the AgFL‐AgNS‐SR_DDTP_7wt.%_ nanocomposite. The *σ* decreases as the DDTP concentration in SR increases beyond 7 wt.% (Figure 4b). The *λ*_B_ is increased to 0.03 and 0.11 eV by further increasing the DDTP concentration to 8 and 10 wt.%, as discussed in Figure 2e. The increased *λ*_B_ hinders the electron tunneling transport, as explained in Equation (1), decreasing *σ*.

The tensile strength of the AgFL‐AgNS‐SR_DDTP_ nanocomposite is also increased with the increase in cross‐linking density (i.e., relative DDTP concentration in SR) as shown in Figure S12 (Supporting Information). The tensile strength is increased from 0.02 to 2.15 MPa as the cross‐linking density increases from 3.67 × 10^−6^ mol cm^−3^ (SR_DDTP_0wt.%_) to 1.64 × 10^−4 ^mol cm^−3^ (SR_DDTP_10wt.%_). Figure S13a (Supporting Information) compares the tensile strength of the pristine SR_DDTP_7wt.%_ specimens synthesized with and without THF peroxide (0.045 m). There is no noticeable difference in tensile strength, indicating the negligible effect of THF peroxide on the mechanical property of the cross‐linked SR itself. In contrast, the tensile strength of the AgFL‐AgNS‐SR_DDTP_7wt.%_ nanocomposite synthesized with THF peroxide (0.045 M) is higher than that of the AgFL‐SR_DDTP_7wt.%_ nanocomposite synthesized without THF peroxide (Figure S13b, Supporting Information). This is due to the uniformly distributed AgNS particles in the nanocomposite.

Figure 4c shows the *σ* of the AgFL‐AgNS‐SR_DDTP_7wt.%_ nanocomposite (AgFL‐AgNS = 40 vol.%) as a function of *δ*_B_. The *λ*_B_ is negligible (0.01 eV) in all the SR_DDTP_7wt.%_ matrix nanocomposites. The *δ*_B_ is controlled by varying THF peroxide concentration as explained in Figure 1f. The *σ* of the AgFL‐SR_DDTP_7wt.%_ nanocomposite is only 5012 S cm^−1^ due to the large *δ*_B_ (1.28 µm) without AgNS particles. The *σ* increases by more than 1030% as *δ*_B_ decreases due to the generation of AgNS particles, resulting in 51 710 S cm^−1^ at *δ*_B_ = 4.1 nm (THF peroxide = 0.045 m). Note that the *σ* does not change significantly when the *δ*_B_ is sufficiently small (<10 nm), demonstrating saturation behavior. The *σ* does not increase further when the THF peroxide concentration is increased beyond 0.045 m (*σ* = 51 945 S cm−¹ at 0.051 m and 51 684 S cm−¹ at 0.062 m). Therefore, 0.045 m is selected as an optimized THF peroxide concentration in this study. Figures 4b,c demonstrate the important effect of both *λ_B_* and *δ*_B_ on *σ*.

Figure 4d shows the normalized current of the AgFL‐SR_DDTP_7wt.%_ nanocomposite (Ag = 40 vol.%, THF peroxide = 0 m) as a function of strain. The *λ_B_* is negligible (0.01 eV), but *δ*_B_ is large (1.28 µm at 0% strain) in the nanocomposite. A low bias voltage of 10 mV is applied in order not to affect the energy barrier alignment with Ag electrods.^[^ ^5^, ^22^ ^]^ The normalized current decreases with increasing strain up to ≈25%, and the nanocomposite with the large *δ*_B_ follows the conventional Ohm'law. Note that *δ*_B_ in the axial direction is further increased with stretching.^[^ ^5^ ^]^ The resistance of conventional conductive materials or nanocomposites is linearly proportional to the length of the specimen.^[^ ^5^ ^]^ This can be explained by Ohm's law or Drude model where the macroscopic resistance is described by microscopic electron scatterings.^[^ ^43^ ^]^ The normalized current drops below the prediction of Ohm's law beyond 25% strain. This could be due to the structural defects and tears generated by stretching. A large tear is observed at 40% strain (Figure 4d, inset; Figure S14, Supporting Information), and the specimen ruptures at 50% strain. The normalized current of the AgFL‐SR_DDTP_2wt.%_ (THF peroxide = 0 m, *δ*_B_ = 1.28 µm, *λ*_B_ = 0.07 eV) nanocomposite is shown as a function of tensile strain (Figure S15, Supporting Information). The transport is diffusive following Ohm's law, regardless of *λ*_B_, when *δ*_B_ is large.

Figure 4e shows the normalized current‐strain characteristics of nanocomposites with small *δ*_B_ values due to the presence of AgNS particles. The normalized current of the AgFL‐AgNS‐SR_DDTP_2wt.%_ nanocomposite (*δ*_B_ = 4.1 nm, THF peroxide = 0.045 m) with a non‐negligible *λ*_B_ (0.07 eV) decreases exponentially with increasing strain. The exponential current decrease precisely matches the prediction of the Simmons approximation model for quantum tunneling Equation (1).^[^ ^5^ ^]^ A more detailed model derivation is provided in Supporting Note 1. Strikingly, the normalized current of the AgFL‐AgNS‐SR_DDTP_7wt.%_ nanocomposite (*δ*_B_ = 8.3 nm, THF peroxide = 0.038 m) with a negligible *λ*_B_ (0.01 eV) is invariant up to 21%. Note that the Simmons approximation model becomes exactly same as the Ohm's law when *λ*_B_ is 0 (Note S1, Supporting Information). The constant current (i.e., invariable resistance) with stretching is achieved when *λ*_B_ is negligible (0.01 eV) and *δ*_B_ is sufficiently small (<10 nm).^[^ ^5^ ^]^ This behavior deviates from the Simmons approximation theory or Ohm's law. There are electron scatterings in the AgFL‐AgNS‐SR_DDTP_7wt.%_ nanocomposite. However, electrons flow in the channel (nanocomposite) without any additional scattering even when the length is increased by stretching. Interestingly, the stretching becomes “diffusive” (following the conventional Ohm's law) when *δ*_B_ is increased beyond 10.3 nm. The current decreases with further stretching (>21.8% strain), which can now be precisely described by Ohm's law. The diffusive stretching occurs, like conventional conductive materials or nanocomposites, when the traveling distance between Ag particles is increased beyond ≈10 nm although *λ*_B_ is still negligible.

As shown in Figure 4f, the current remains constant until the AgFL‐AgNS‐SR_DDTP_7wt.%_ nanocomposite is stretched up to 53% strain (applied voltage = 10 mV). This is achieved by decreasing the initial *δ*_B_ at 0% strain to 4.1 nm (THF peroxide = 0.045 m). The *λ*_B_ is also negligible (0.01 eV). The corresponding invariable resistance up to 53% strain is shown in Figure S16 (Supporting Information). The *δ*_B_ is increased to 6.3 nm at 53% strain. The rapid decrease in current beyond 53% strain is due to the specimen breakage, rather than the failure in strain‐independent resistance, as shown in optical microscopic images (Figure 4f, inset). The nanocomposite completely ruptures at 57% strain. The SEM images show interfacial debonding around a tear (Figure S17, Supporting Information). The stretching cycle test is carried out with a maximum strain of 40%. The normalized current of the AgFL‐AgNS‐SR_DDTP_7wt.%_ nanocomposite (AgFL‐AgNS = 40 vol.%, THF peroxide = 0.045 m) at 40% strain remains constant during 300 stretching cycles (Figure S18, Supporting Information). However, the nanocomposite does not return to its initial length, when stress is released, although the high conductance is maintained at 40% strain. The mechanical reversibility of the nanocomposite needs to be improved in the future. Note that other elastomers, such as Ecoflex, could potentially achieve invariant conductance at a higher strain range. This needs to be investigated further in the future since nanocomposites with different matrix polymers need to be optimized again. Different polymers have different gap state energy levels, and therefore different barrier heights.

The unique electrical transport behavior of the AgFL‐AgNS‐SR_DDTP_7wt.%_ nanocomposite with a *δ*_B_ of 4.1 nm is further supported by the magnetoresistance (*R*_xx_) of the nanocomposite (Figure 4g). Here, the origin of the constant resistance with stretching is attributed to the absence of additional scattering between AgNS particles in a stretched channel. Given that the stretching occurs in the polymer matrix, the absence of additional scattering indicates that the interparticle distance between AgNS particles during stretching is still shorter than the mean free path of the electrons in the nanocomposite. Then, electrons can keep their wavefunctions without additional scatterings between AgNS particles in the transport, which can be perturbed by a vertical magnetic field and can be observed as negative magnetoresistance. Thus, the negative magnetoresistance with a *δ*_B_ of 4.12 nm, shown as a blue curve in Figure 4g, can be explained by coherent transport between AgNS particles, which results in the constant resistance with stretching.

To estimate the length scale that can avoid additional stretching‐induced scattering of electrons and generate negative magnetoresistance in the nanocomposite, we fitted the blue curve in Figure 4g by the Hikami‐Larkin‐Nagaoka equation.^[^ ^44^ ^]^

(2) $$\rho_{\left(B\right)}=\rho_{\left(0\right)}\;-\frac{e^{2}{R_{s}}^{2}}{2\pi^{2}\hbar }\left[\psi \left(\frac{1}{2}+\frac{B_{i}}{B}\right)+ln\frac{B}{B_{i}}\right]$$

where *ρ_(B)_* is the resistivity at a magnetic field *B*, *R*_s_ is the sheet resistance, *ψ* is the digamma function, *ħ* is reduced Planck's constant, $B_{i}=\frac{\hbar }{4el_{i}^{2}}$, and *l*_i_ is the phase coherent length. A phase coherent length of 20 nm is obtained by the fitting of the negative magnetoresistance, which is longer than the interparticle distance between AgNS particles (*δ*_B_ = 4.1 nm).

Note that typical diffusive transport in a longer polymer matrix channel (involving numerous scatterings) exhibits negligible or positive magnetoresistance, which is shown as a black curve in Figures 4g and S19 (Supporting Information). As a control experiment, we measured the magnetoresistance of the nanocomposite with a *δ*_B_ of 20.3 nm (AgFL‐AgNS‐SR_DDTP_7wt.%_, THF peroxide = 0.023 M), where the interparticle distance is comparable to the length scale derived from the fitting of the blue curve in Figure 4g. The nanocomposite with a longer AgNS interparticle distance does not show negative magnetoresistance, which confirms the role of the interparticle distance in the magneto‐transport and strain‐invariant resistance. The AgFL‐SR_DDTP_7wt.%_ nanocomposite with a substantially greater *δ*_B_ (1.28 µm) also does not show the negative magnetoresistance Figure S19 (Supporting Information), while the *R*_xx_ values are significantly higher than those from the nanocomposite with a shorter *δ*_B_. This further demonstrates the importance of small *δ*_B_ for the strain‐invariant resistance of the nanocomposite.

Figure 4h shows the energy band alignment diagram of the Ag, SR_DDTP_2wt.%_, and SR_DDTP_7wt.%_. The *φ*_Ag_ (4.70 eV) and *τ*_SR_ (4.63 eV for SR_DDTP_2wt.%_ and 4.71 eV for SR_DDTP_7wt.%_), measured by KPFM, are written in blue. The corresponding *λ*_B_ (= *φ*_Ag_ – *τ*_SR_) values obtained by KPFM are also indicated in blue. Note that the Fermi energy level (*E*_F_) is aligned in the diagram, and the polymer bandgap is obtained from the absorption spectrum and Tauc plot analysis (Figures S20 and S21, Supporting Information). The *λ*_B_ can also be obtained from the Simmons approximation model fitting process (see Note S1, Supporting Information for details).^[^ ^5^ ^]^ The *λ*_B_ obtained from the Simmons model fitting of the AgFL‐AgNS‐SR_DDTP_2wt.%_ nanocomposite, denoted using a green dash line, precisely matches the *λ*_B_ obtained by KPFM. The barrier analysis conducted by two different methods further provides reliability.^[^ ^5^ ^]^ The *λ*_B_ cannot be obtained for the AgFL‐AgNS‐SR_DDTP_7wt.%_ nanocomposite by the Simmons model fitting analysis, because the experimental data deviate from the theory (Figure 4e).

The *σ* at 0% strain and normalized resistance change (*ΔR*/*R*_0_) at 50% strain of the AgFL‐AgNS‐SR_DDTP_7wt.%_ nanocomposite (AgFL‐AgNS = 40 vol.%, *δ*_B_ = 4.1 nm, *λ*_B_ = 0.01 eV, solid red star) are compared with those of polymer matrix nanocomposites in literature (open symbols) as shown in Figure 4i.^[^ ^5^, ^8^, ^13^, ^16^, ^17^, ^20^, ^45^, ^46^, ^47^, ^48^, ^49^, ^50^ ^]^ A more detailed information is provided in Table S1 (Supporting Information). Note that only intrinsically stretchable nanocomposites are compared, excluding the effect of external geometrical modifications such as kirigami‐inspired structures, serpentine designs, and surface wrinkling/buckling techniques.^[^ ^9^, ^10^, ^11^, ^12^ ^]^ The nanocomposite from our previous study (half‐filled blue star) showed an invariable resistance up to 30% strain.^[^ ^5^ ^]^ However, the *σ* was very small (≈12 S cm^−1^) due to the uncross‐linked matrix polymer, and the specimen ruptured at 30% strain.^[^ ^5^ ^]^ Strikingly, the *σ* (51 710 S cm^−1^) of the cross‐linked AgFL‐AgNS‐SR_DDTP_7wt.%_ nanocomposite is increased by more than 3 orders of magnitude, compared with our previous work,^[^ ^5^ ^]^ due to the removal of voids and precisely controlled *λ*_B_. The rupture strain is also increased to 53%. The AgFL‐AgNS‐SR_DDTP_7wt.%_ nanocomposite provides the very high *σ* and negligible *ΔR*/*R*_0_ (at 50% strain) compared with other nanocomposites in literature.

## 3 Conclusion

The electron transport of conductive nanocomposites strongly depends on *δ*_B_ and *λ*_B_. We have developed the methods to systematically manipulate the two critical parameters. The *δ*_B_ can be precisely modulated by the in situ generated small AgNS particles using THF peroxide, and *λ*_B_ can be tuned by the gap state energy level of SR_DDTP_ using cross‐linkers. When *δ*_B_ is large (e.g., 1.28 µm), the transport in our nanocomposites is described by the diffusive model‐based on Ohm's law, regardless of *λ*_B_. On the other hand, the transport is dominated by quantum tunneling, following the Simmons approximation theory, when *δ*_B_ is small and *λ*_B_ is non‐negligible (e.g., AgFL‐AgNS‐SR_DDTP_2wt.%_, *δ*_B_ = 4.1 nm, *λ*_B_ = 0.07 eV). The strain‐invariant resistance is observed when *λ*_B_ is negligible and *δ*_B_ is small (e.g., AgFL‐AgNS‐SR_DDTP_7wt.%_, *δ*_B_ = 4.1 nm, *λ*_B_ = 0.01 eV). The stretching‐induced additional electron scattering is absent up to *δ*_B_ = ≈10 nm in the nanocomposite, resulting in a strain‐invariant resistance. Furthermore, the *σ* (51 710 S cm^−1^) of the cross‐linked AgFL‐AgNS‐SR_DDTP_7wt.%_ nanocomposite is increased by more than 4 orders of magnitude, compared with the uncross‐linked AgFL‐AgNS‐SR_DDTP_0wt.%_ nanocomposite (Ag = 40 vol.%) with a high *λ*_B_ (0.14 eV). The strong C‐C covalent bonds, formed by cross‐linking, also remove voids and significantly increase mechanical strength. The novel strain‐invariant resistance mechanism, supported by the KPFM measurement, Simmons approximation theory, and magnetoresistance analysis, may be useful for stretchable conductive electrodes with an unchanging resistance over large strain.

## 4 Experimental Section

### Synthesis of the AgFL‐AgNS‐SR_DDTP_ and AgFL‐SR_DDTP_ Nanocomposites

The THF without a BHT inhibitor (Sigma–Aldrich, 401757) was air bubbled for ≈72 h to prepare THF peroxide.^[^ ^5^ ^]^ The air bubbling process was carried out using an air pump and a flow regulator (air flow rate = ≈5 mL s^−1^). The SR (KCC Silicon corporation, SH0010U, 15 wt.%) was dissolved in THF with a BHT inhibitor (Sigma–Aldrich, 186562). The 2,5‐dimethyl‐2,5‐di(tert‐butylperoxy) hexane (KCC Silicon corporation, 8 wt.%) was also dissolved in hexane (Sigma–Aldrich, 178918) to prepare the DDTP solution. In the next step, AgFLs (Metalor, SA‐31812) were dispersed in the mixture of SR (2 g) and DDTP solutions with additional THF peroxide (15 mL, 0.01‐0.045 m) by tip sonication (420 W, 10 min). The mixture was further stirred for ≈45 min (300 rpm) to generate AgNS particles. The mixture was then drop‐casted and dried overnight at room temperature. Finally, the specimen was hot‐pressed at 170 °C (3 MPa, 10 min) followed by curing (200 °C, 4 h) to obtain the cross‐linked AgFL‐AgNS‐SR_DDTP_ nanocomposite. The amount of AgFLs (35–44 vol.%) and the relative concentration of DDTP in SR (0‐10 wt.%) were precisely controlled in the final nanocomposite, after evaporation of the solvent. The AgFL‐SR_DDTP_ nanocomposite was synthesized using THF with a BHT inhibitor. The other procedures were identical. The cross‐linked SR_DDTP_ was prepared by mixing SR and DDTP solutions, without AgFLs and THF peroxide.

### Characterization

The microstructure was investigated by HRTEM (JEOL, Cs‐corrected JEM‐ARM 200F) and SEM (JEOL, JSM‐7600F). The electrical conductivity of the nanocomposites was measured by the four‐point probe in‐line method using a laboratory‐built device.^[^ ^6^, ^18^ ^]^ The resistance was measured by a current source (Keithley 6221) and a nanovoltmeter (Keithley 2182A). The distance between tungsten probes was 1 mm. A detailed description of the geometry calibration was provided elsewhere.^[^ ^18^, ^51^ ^]^ The stress‐strain characteristic was measured by a universal testing machine (Instron, 3343). The strain‐dependent current change of the nanocomposite was measured using a direct current power supply (Agilent, E3648A) and a data acquisition software (Keysight BenchVue).^[^ ^5^ ^]^ The surface potential was measured by KPFM (Nanonavi, E‐sweep) using a gold‐coated silicon tip.^[^ ^5^, ^38^ ^]^ The AgFLs dispersed in THF with a BHT inhibitor were drop‐casted on a silicon wafer, and the work function was directly measured on the AgFLs.^[^ ^5^ ^]^ The smooth and thin SR_DDTP_ films (DDTP = 0–10 wt.%) were transferred on a silicon wafer for the KPFM measurement. The cross‐section of the nanocomposite was polished using polishing sheets (grit 2000 and grit 15000, 1 minute) and microfiber cloth for the KPFM area mapping analysis. The magnetoresistance was measured using a cryostat (Oxford instruments, temperature range = 1.5–300 Κ, magnetic field range = 0–12 T).

### Statistical Analysis

Statistical values, such as mean and standard deviations, were provided. Three specimens were tested at each condition in Figures 1, 2, and 4a–f. The error bar indicates the standard deviation of the data.

## Conflict of Interest

The authors declare no conflict of interest.

## Author Contributions

C.M.A., H.Y., and S.B. conceived and designed the experiments, which were carried out by C.M.A., J.J., S.C., and M.K.M. J.J., and H.Y. carried out the magnetoresistance measurements. C.M.A., H.Y., and S.B. wrote the paper. All authors contributed to data analysis and scientific discussion.

## Supporting information



Supporting Information

## Data Availability

The data that support the findings of this study are available from the corresponding author upon reasonable request.

## References

[1] S. Gong , X. Zhang , X. A. Nguyen , Q. Shi , F. Lin , S. Chauhan , Z. Ge , W. Cheng , Nat. Nanotechnol. 2023, 18, 889. 37106048 10.1038/s41565-023-01383-6

[2] S. Lee , S. Franklin , F. A. Hassani , T. Yokota , M. O. G. Nayeem , Y. Wang , R. Leib , G. Cheng , D. W. Franklin , T. Someya , Science 2020, 370, 966. 33214278 10.1126/science.abc9735

[3] S. Choi , S. I. Han , D. Jung , H. J. Hwang , C. Lim , S. Bae , O. K. Park , C. M. Tschabrunn , M. Lee , S. Y. Bae , J. W. Yu , J. H. Ryu , S.‐W. Lee , K. Park , P. M. Kang , W. B. Lee , R. Nezafat , T. Hyeon , D.‐H. Kim , Nat. Nanotechnol. 2018, 13, 1048. 30104619 10.1038/s41565-018-0226-8

[4] H. Xu , S. Wu , Y. Liu , X. Wang , A. K. Efremov , L. Wang , J. S. McCaskill , M. Medina‐Sánchez , O. G. Schmidt , Nat. Nanotechnol. 2024, 19, 494. 38172430 10.1038/s41565-023-01567-0 PMC11026159

[5] C. M. Ajmal , S. Cha , W. Kim , K. P. Faseela , H. Yang , S. Baik , Sci. Adv. 2022, 8, eabn3365. 35960794 10.1126/sciadv.abn3365 PMC9374331

[6] D. Suh , K. P. Faseela , W. Kim , C. Park , J. G. Lim , S. Seo , M. K. Kim , H. Moon , S. Baik , Nat. Commun. 2020, 11, 2252. 32382034 10.1038/s41467-020-15709-8 PMC7206115

[7] K. P. Faseela , C. M. Ajmal , S. Cha , S. Baik , Adv. Funct. Mater. 2023, 33, 2304776.

[8] N. Matsuhisa , D. Inoue , P. Zalar , H. Jin , Y. Matsuba , A. Itoh , T. Yokota , D. Hashizume , T. Someya , Nat. Mater. 2017, 16, 834. 28504674 10.1038/nmat4904

[9] H. Liu , H. Li , Z. Wang , X. Wei , H. Zhu , M. Sun , Y. Lin , L. Xu , Adv. Mater. 2022, 34, 2207350. 10.1002/adma.202207350 36222392

[10] H. Choi , Y. Luo , G. Olson , P. Won , J. H. Shin , J. Ok , Y. J. Yang , T.‐i. Kim , C. Majidi , Adv. Funct. Mater. 2023, 33, 2301388.

[11] S. Jang , C. Kim , J. J. Park , M. L. Jin , S. J. Kim , O. O. Park , T.‐S. Kim , H.‐T. Jung , Small 2018, 14, 1702818. 10.1002/smll.201702818 29280274

[12] G. Lee , M. Zarei , Q. Wei , Y. Zhu , S. G. Lee , Small 2022, 18, 2203491. 10.1002/smll.202203491 36047645

[13] S. Liu , D. S. Shah , R. Kramer‐Bottiglio , Nat. Mater. 2021, 20, 851. 33603186 10.1038/s41563-021-00921-8

[14] S. Veerapandian , W. Jang , J. B. Seol , H. Wang , M. Kong , K. Thiyagarajan , J. Kwak , G. Park , G. Lee , W. Suh , I. You , M. E. Kılıç , A. Giri , L. Beccai , A. Soon , U. Jeong , Nat. Mater. 2021, 20, 533. 33398123 10.1038/s41563-020-00863-7

[15] C. S. Boland , U. Khan , G. Ryan , S. Barwich , R. Charifou , A. Harvey , C. Backes , Z. Li , M. S. Ferreira , M. E. Möbius , R. J. Young , J. N. Coleman , Science 2016, 354, 1257. 27940866 10.1126/science.aag2879

[16] D. Jung , Y. Kim , H. Lee , S. Jung , C. Park , T. Hyeon , D.‐H. Kim , Adv. Mater. 2023, 35, 2303458. 10.1002/adma.202303458 37591512

[17] M. Gu , W.‐J. Song , J. Hong , S. Y. Kim , T. J. Shin , N. A. Kotov , S. Park , B.‐S. Kim , Sci. Adv. 2019, 5, eaaw1879. 31360766 10.1126/sciadv.aaw1879 PMC6660205

[18] K.‐Y. Chun , Y. Oh , J. Rho , J.‐H. Ahn , Y.‐J. Kim , H. R. Choi , S. Baik , Nat. Nanotechnol. 2010, 5, 853. 21113161 10.1038/nnano.2010.232

[19] R. Ma , S. Kwon , Q. Zheng , H. Y. Kwon , J. I. Kim , H. R. Choi , S. Baik , Adv. Mater. 2012, 24, 3344. 22628086 10.1002/adma.201201273

[20] D. Jung , C. Lim , C. Park , Y. Kim , M. Kim , S. Lee , H. Lee , J. H. Kim , T. Hyeon , D.‐H. Kim , Adv. Mater. 2022, 34, 2200980. 10.1002/adma.202200980 35388541

[21] G. Lubineau , A. Mora , F. Han , I. N. Odeh , R. Yaldiz , Comput. Mater. Sci. 2017, 130, 21.

[22] J. G. Simmons , J. Appl. Phys 1963, 34, 1793.

[23] N. N. Jason , M. D. Ho , W. Cheng , J. Mater. Chem. C 2017, 5, 5845.

[24] J. Li , J.‐K. Kim , Compos. Sci. Technol. 2007, 67, 2114. 10.1016/j.compscitech.2007.04.016 PMC7127559 32287828

[25] J. G. Simmons , J. Phys. D: Appl. Phys. 1971, 4, 613.

[26] N. Matthews , M. J. Hagmann , A. Mayer , J. Appl. Phys. 2018, 123, 136101.

[27] K. Mistry , M. Yavuz , K. P. Musselman , J. Appl. Phys. 2017, 121, 184504.

[28] J. G. Simmons , J. Phys. Chem. Solids 1971, 32, 2581.

[29] T. Mizutani , Y. Takai , T. Osawa , M. Ieda , J. Phys. D: Appl. Phys. 1976, 9, 2253.

[30] Y. Wang , S. Nasreen , D. Kamal , Z. Li , C. Wu , J. Huo , L. Chen , R. Ramprasad , Y. Cao , ACS Appl. Mater. Interfaces 2021, 13, 46142. 34520160 10.1021/acsami.1c12854

[31] S.‐H. Lee , S. W. Lee , T. Oh , S. H. Petrosko , C. A. Mirkin , J.‐W. Jang , Nano Lett. 2018, 18, 109. 29140713 10.1021/acs.nanolett.7b03540

[32] H. Matsubara , S. Suzuki , S. Hirano , Org. Biomol. Chem. 2015, 13, 4686. 25798812 10.1039/c5ob00012b

[33] E.‐S. Park , J. Appl. Polym. Sci. 2008, 110, 1723.

[34] J. B. Class , R. P. Grasso , Rubber Chem. Technol. 1993, 66, 605.

[35] J. Schweitzer , S. Merad , G. Schrodj , F. Bally‐Le Gall , L. Vonna , J. Chem. Educ. 2019, 96, 1472.

[36] P. J. Flory , J. Rehner Jr. , J. Chem. Phys. 1943, 11, 521.

[37] R. A. Orwoll , P. A. Arnold , in Physical Properties of Polymers Handbook (Ed: J. E. Mark ), Springer, New York 2007.

[38] B. Kang , Y. Kim , W. J. Yoo , C. Lee , Small 2018, 14, 1802593. 10.1002/smll.201802593 30256520

[39] H. Lee , W. Lee , J. H. Lee , D. S. Yoon , J. Nanomater. 2016, 2016, 4209130.

[40] A. Kahn , Mater. Horiz. 2016, 3, 7.

[41] Z. Yan , K. Yang , Y. Zhang , S. Wang , J. Li , J. Mater. Sci. Mater. Electron. 2019, 30, 20605.

[42] K. Yang , Y. Liu , Z. Yan , Y. Tian , Y. Liu , Z. Jing , J. Li , S. Li , Materials 2020, 13, 3935. 32899561 10.3390/ma13183935 PMC7559297

[43] N. W. Ashcroft , N. D. Mermin , Solid State Physics, Saunders College Publishing, New York 1976.

[44] R. L. Samaraweera , H. C. Liu , B. Gunawardana , A. Kriisa , C. Reichl , W. Wegscheider , R. G. Mani , Sci. Rep. 2018, 8, 10061. 29968817 10.1038/s41598-018-28359-0 PMC6030049

[45] P. A. Lopes , D. F. Fernandes , A. F. Silva , D. G. Marques , A. T. de Almeida , C. Majidi , M. Tavakoli , ACS Appl. Mater. Interfaces 2021, 13, 14552. 33689286 10.1021/acsami.0c22206

[46] L. Zhu , Y. Wang , D. Mei , W. Ding , C. Jiang , Y. Lu , ACS Appl. Mater. Interfaces 2020, 12, 31725. 32569461 10.1021/acsami.0c09653

[47] D. Jung , C. Lim , H. J. Shim , Y. Kim , C. Park , J. Jung , S. I. Han , S.‐H. Sunwoo , K. W. Cho , G. D. Cha , D. C. Kim , J. H. Koo , J. H. Kim , T. Hyeon , D.‐H. Kim , Science 2021, 373, 1022. 34446604 10.1126/science.abh4357

[48] C. J. Thrasher , Z. J. Farrell , N. J. Morris , C. L. Willey , C. E. Tabor , Adv. Mater. 2019, 31, 1903864. 10.1002/adma.201903864 31403234

[49] J. Wang , G. Cai , S. Li , D. Gao , J. Xiong , P. S. Lee , Adv. Mater. 2018, 30, 1706157. 10.1002/adma.201706157 29512208

[50] S.‐H. Sunwoo , S. I. Han , D. Jung , M. Kim , S. Nam , H. Lee , S. Choi , H. Kang , Y. S. Cho , D.‐H. Yeom , M.‐J. Cha , S. Lee , S.‐P. Lee , T. Hyeon , D.‐H. Kim , ACS Nano 2023, 17, 7550. 37039606 10.1021/acsnano.2c12659

[51] F. M. Smits , Bell Syst. Tech. J. 1958, 37, 711.

