# Supplementary material for: Absence of Additional Stretching‐Induced Electron Scattering in Highly Conductive Cross‐linked Nanocomposites with Negligible Tunneling Barrier Height and Width
Source: Adv Sci (Weinh). 2024 Oct 28;11(48):2409337. doi: 10.1002/advs.202409337 (PMC11672263; doi:10.1002/advs.202409337)
Supplement: Supplementary file 1 — Supporting Information [file ADVS-11-2409337-s001.docx]

**Supporting Information**

**Absence of Additional Stretching-Induced Electron Scattering in Highly Conductive Crosslinked Nanocomposites with Negligible Tunneling Barrier Height and Width**

C. Muhammed Ajmal, Juyeong Jeong, Seongsu Cheon, M. K. Majee, Heejun Yang*, and Seunghyun Baik*

C.M. Ajmal, M.K. Majee, S. Baik

Center for Nanotubes and Nanostructured Composites

Sungkyunkwan University

Suwon, 16419, Republic of Korea

J. Jeong, H. Yang

Department of Physics

Korea Advanced Institute of Science and Technology (KAIST)

Daejeon 34141, Republic of Korea.

S. Cheon, S. Baik

School of Mechanical Engineering

Sungkyunkwan University

Suwon, 16419, Republic of Korea

E-mail: [h.yang@kaist.ac.kr](mailto:h.yang@kaist.ac.kr), [sbaik@skku.edu](mailto:sbaik@skku.edu)

**Keywords:** Resistance, crosslinked nanocomposites, tunneling, barrier height, barrier width

**
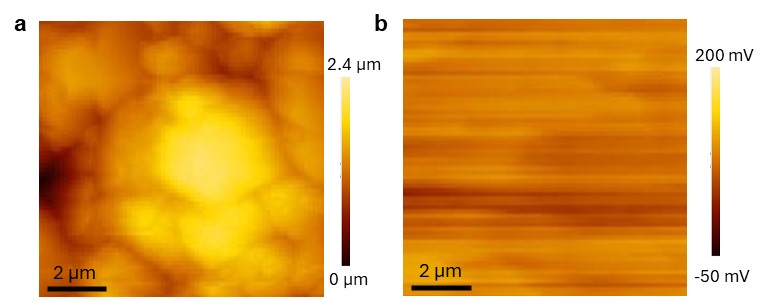
**

**Figure S1.** The KPFM measurements of AgFLs. **a** Topography. **b** Contact potential difference.


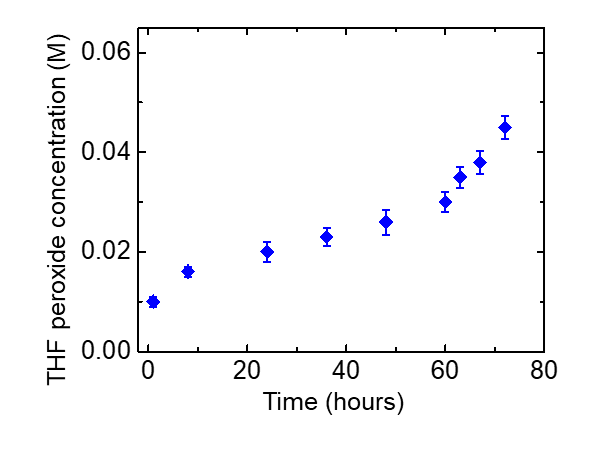


**Figure S2.** The THF peroxide concentration is measured by the iodometric titration method as a function of air bubbling time.^[1,2]^ The air flow rate is ~ 5 mL/s.


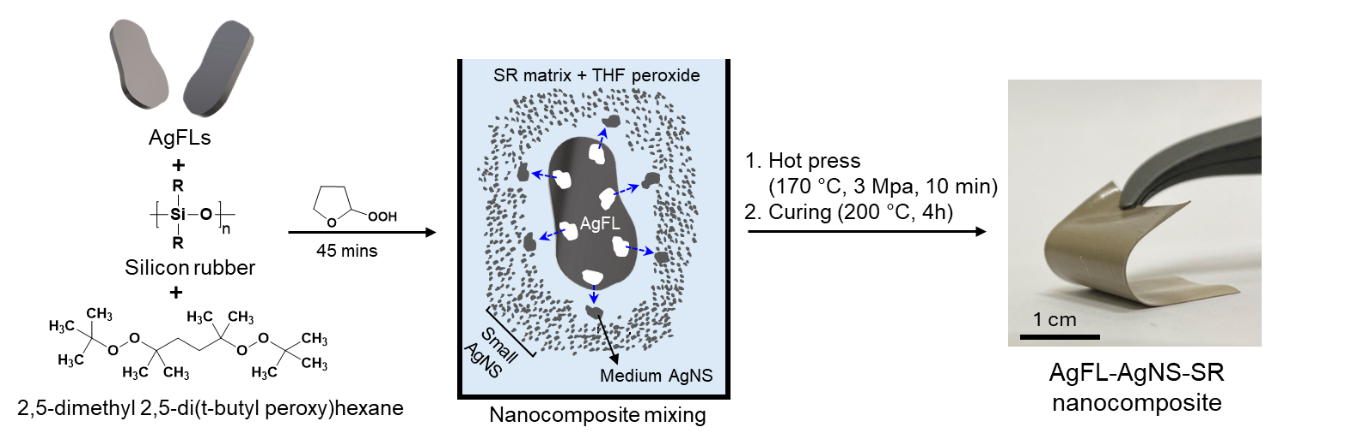


**Figure S3.** Schematic of the synthesis process of the crosslinked AgFL-AgNS-SR_DDTP_ nanocomposite.


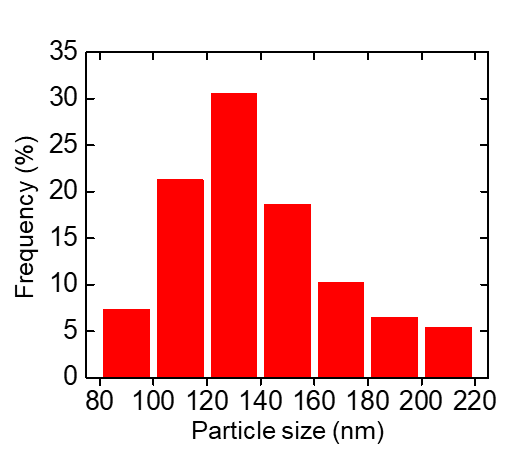


**Figure S4.** The size distribution of medium AgNS particles in the AgFL-AgNS-SR_DDTP_7wt%_ nanocomposite (AgNF-AgNS = 40 vol%). The average particle size is ~139 nm.


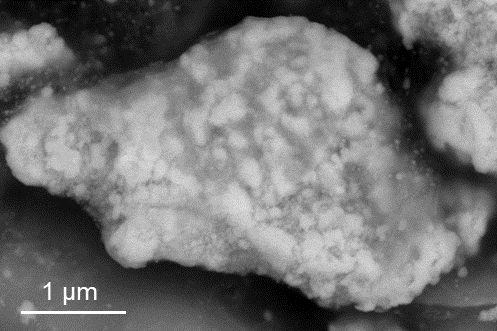


**Figure S5.** SEM image of the in-situ etched surface of AgFLs in the AgFL-AgNS-SR_DDTP_7wt%_ nanocomposite (AgFL-AgNS = 40 vol%).


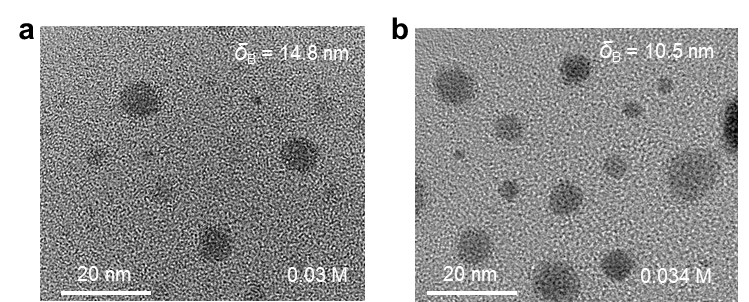


**Figure S6.** HRTEM images of small AgNS particles in the AgFL-AgNS-SR_DDTP_7wt%_ nanocomposite (AgFL-AgNS = 40 vol%). **a** THF peroxide concentration = 0.03 M. **b** THF peroxide concentration = 0.034 M.

**
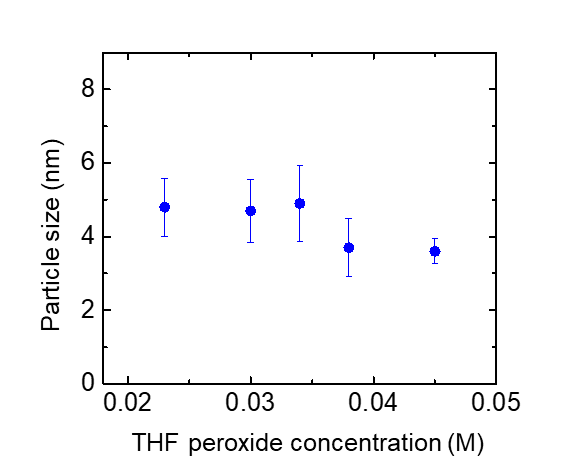
**

**Figure S7.** The size of small AgNS particles is shown as a function of the THF peroxide concentration (AgFL-AgNS-SR_DDTP_7wt%_, AgFL-AgNS = 40 vol%). The average particle sizes are 4.8, 4.7, 4.9, 3.7, and 3.6 nm at THF peroxide concentrations of 0.023, 0.03, 0.034, 0.038 and 0.045 M, respectively.


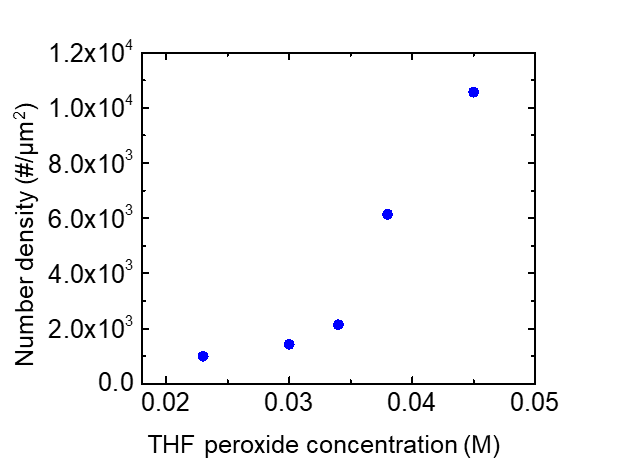


**Figure S8.** The number density of small AgNS particles is shown as a function of the THF peroxide concentration (AgFL-AgNS-SR_DDTP_7wt%_, AgFL-AgNS = 40 vol%).


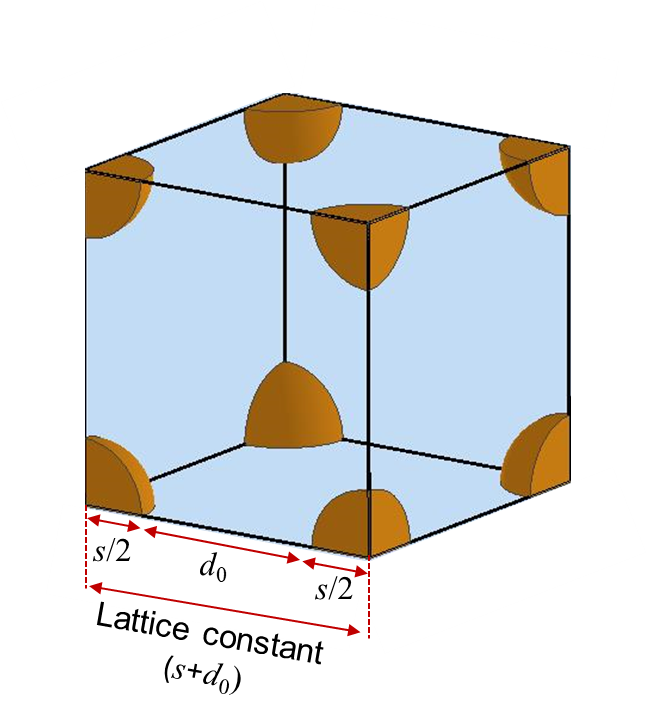


**Figure S9.** Schematic of the simple cubic structure of AgNS particles.^[1]^ The lattice constant is the sum of the average particle size (*s*) and interparticle distance (*d_0_*).


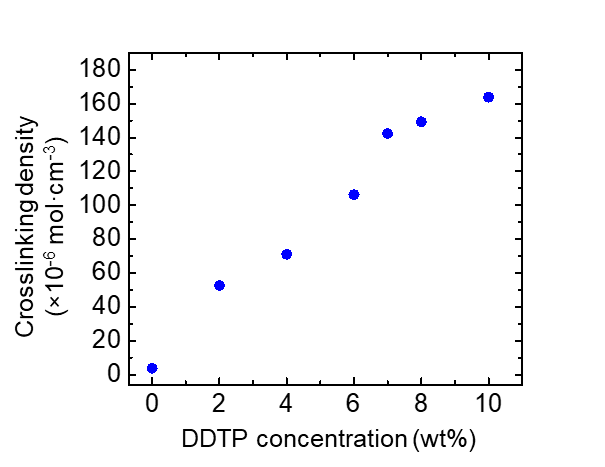


**Figure S10.** The crosslinking density is shown as a function of the DDTP concentration in SR.

The crosslinking density is measured by the equilibrium swelling method.^[3]^ The swelling test is carried out using toluene following the ASTM-D2765-95 standard. The specimens with different DDTP concentrations (0-10 wt%) are weighed and soaked in toluene for 48 hours at room temperature. The specimens are then taken out of toluene and allowed to attain equilibrium weight. The swelling degree (SD) is expressed by Equation 1.^[3]^

SD = $\frac{{{(m}_{0}}/{\rho_{SR})+ {(m-m_{0})}/{\rho_{t}}}}{{m_{0}}/{\rho_{SR}}}$ (1)

where *m*_0_ and *m* are the specimen’s masses before and after soaking in toluene, respectively. The *ρ*_SR_ and *ρ*_t_ are the densities of SR and toluene, respectively. The crosslinking density is then calculated using the Flory–Rehner equation.^[4]^

Crosslinking density = $-\frac{\ln\left( 1-V_{SR} \right)+V_{SR}+\beta{V_{SR}}^{2}}{V_{t}({V_{SR}}^{1/3}-0.5V_{SR})}$ (2)

where *V*_SR_ = 1/SD, *V*_t_ is the molar volume of toluene (106.3 mL/mol), and *β* is the Flory-Huggins solvent-polymer interaction parameter for toluene and SR (0.445).^[3-5]^


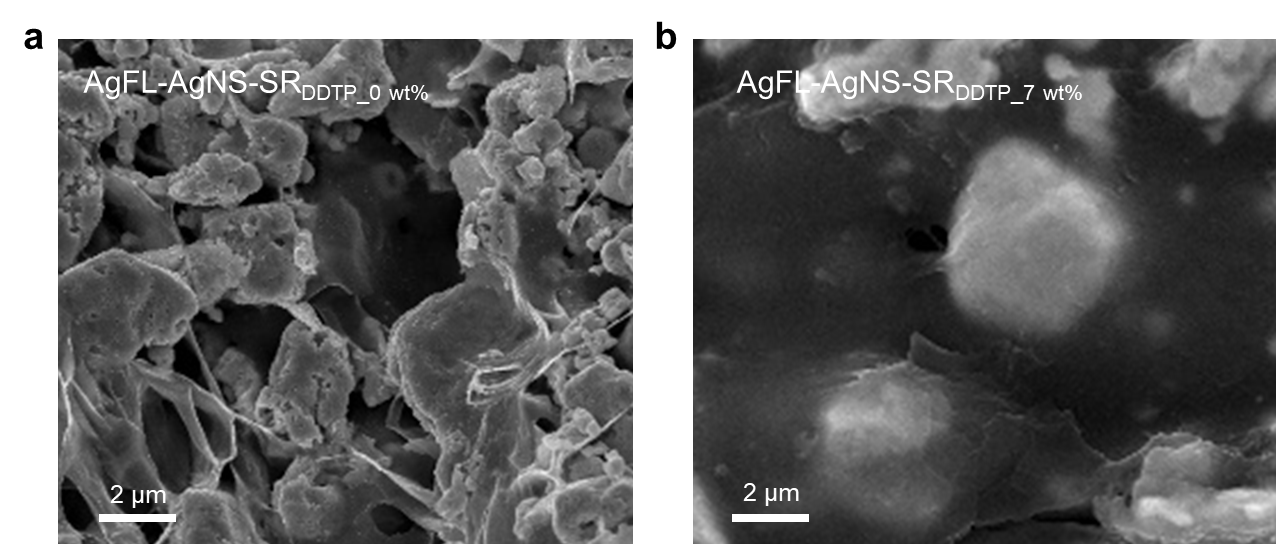


**Figure S11.** The magnified cross-sectional SEM images. **a** AgFL-AgNS-SR_DDTP_0wt%_. **b** AgFL-AgNS-SR_DDTP_7wt%_.


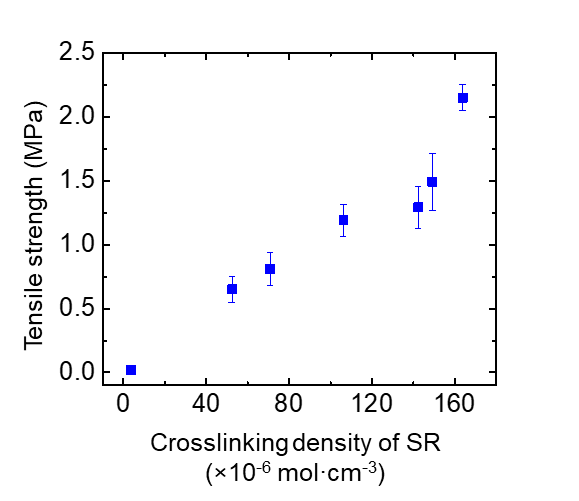


**Figure S12.** The tensile strength of the AgFL-AgNS-SR_DDTP_ nanocomposite (AgFL-AgNS = 40 vol%) is shown as a function of the crosslinking density of SR.

**
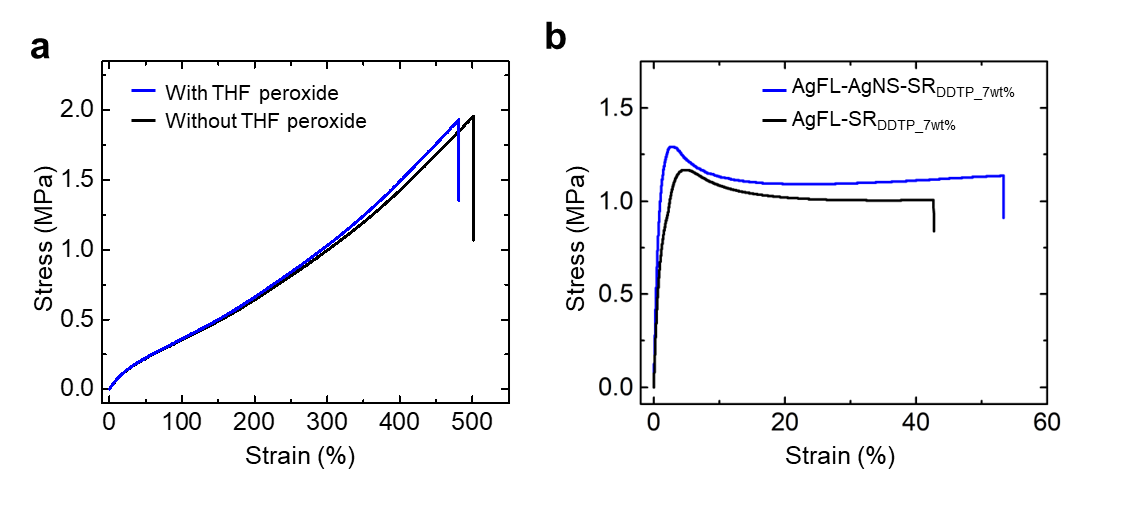
**

**Figure S13.** Tensile stress-strain characteristics. **a** The pristine SR_DDTP_7wt%_ specimens are synthesized with and without THF peroxide (0.045 M). The Ag particles are not incorporated in the specimens. **b** The AgFL-AgNS-SR_DDTP_7wt%_ and AgFL-SR_DDTP_7wt%_ nanocomposites (Ag = 40 vol%) are synthesized with and without THF peroxide (0.045 M), respectively.


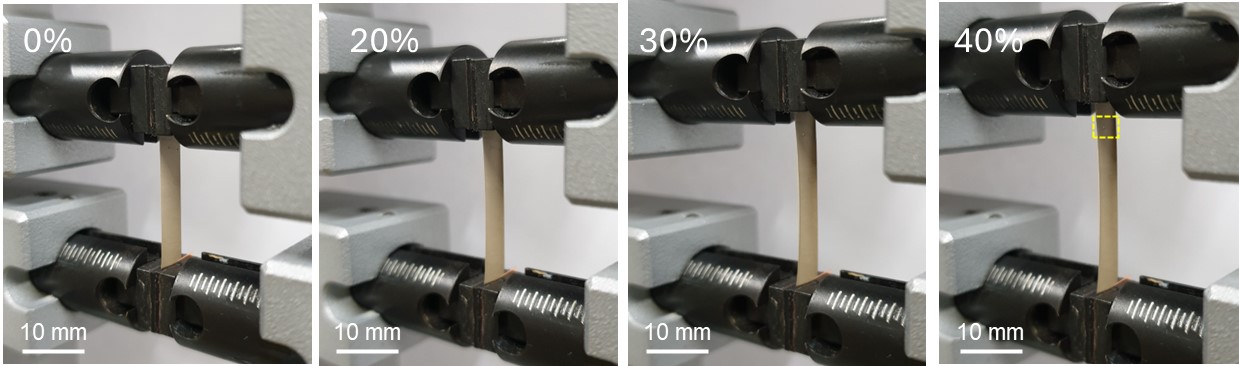


**Figure S14.** The optical images of the stretching process of the AgFL-SR_DDTP_7wt%_ nanocomposite (AgFL = 40 vol%). A tear is observed at 40 % strain (denoted by a yellow square).


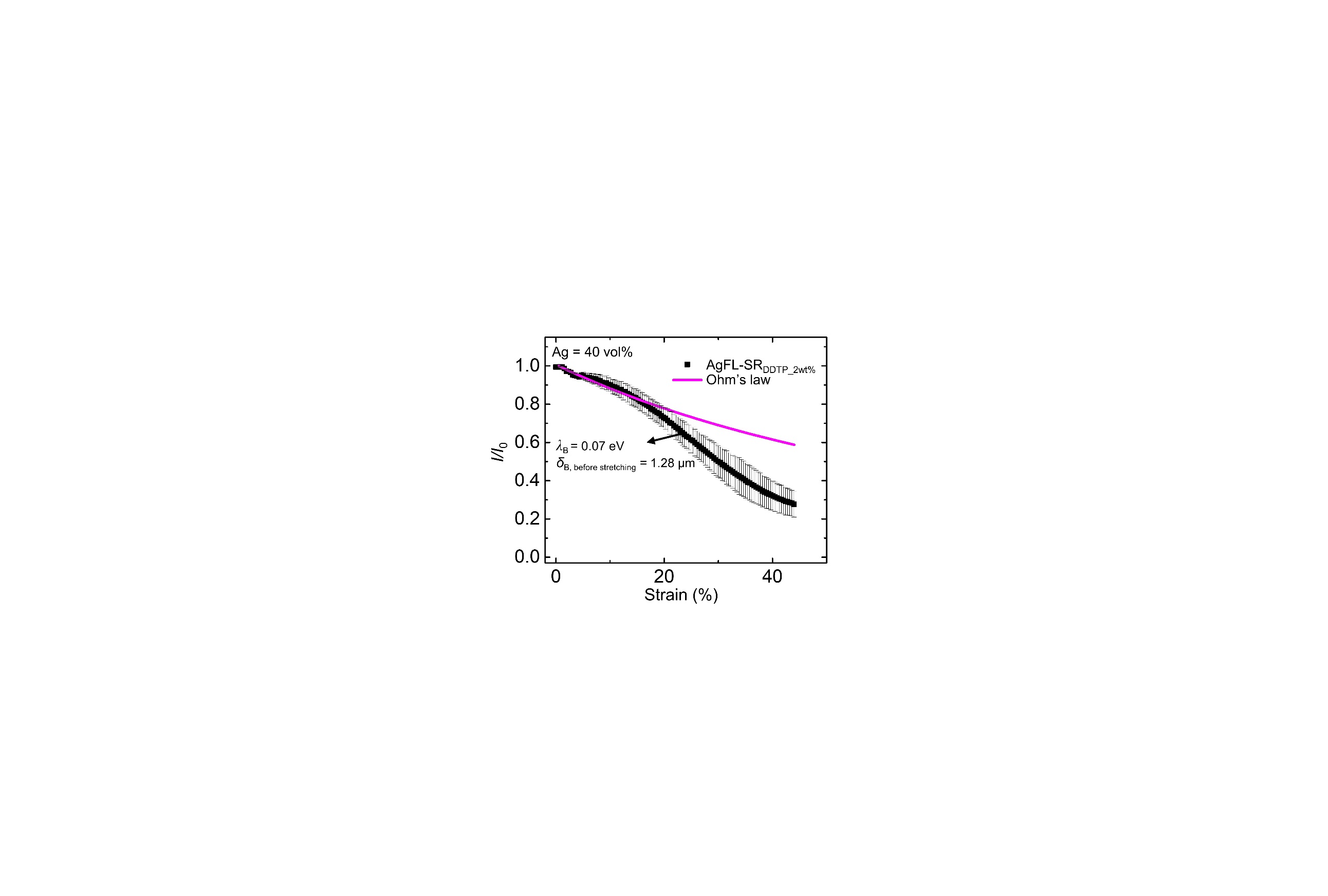


**Figure S15.** The normalized current of the AgFL-SR_DDTP_2wt%_ (THF peroxide = 0 M) nanocomposite as a function of tensile strain (applied voltage = 10 mV, crosshead speed = 1 mm/min). The theoretical prediction by Ohm’s law, assuming constant resistivity, is also shown.


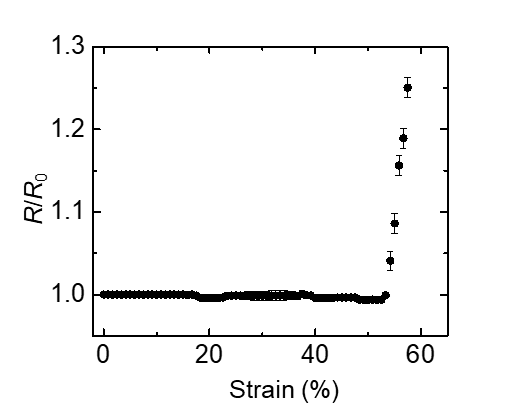


**Figure S16.** The normalized resistance of the AgFL-AgNS-SR_DDTP_7wt%_ nanocomposite (AgFL-AgNS = 40 vol%, THF peroxide = 0.045 M) as a function of tensile strain (applied voltage = 10 mV, crosshead speed = 1 mm/min).


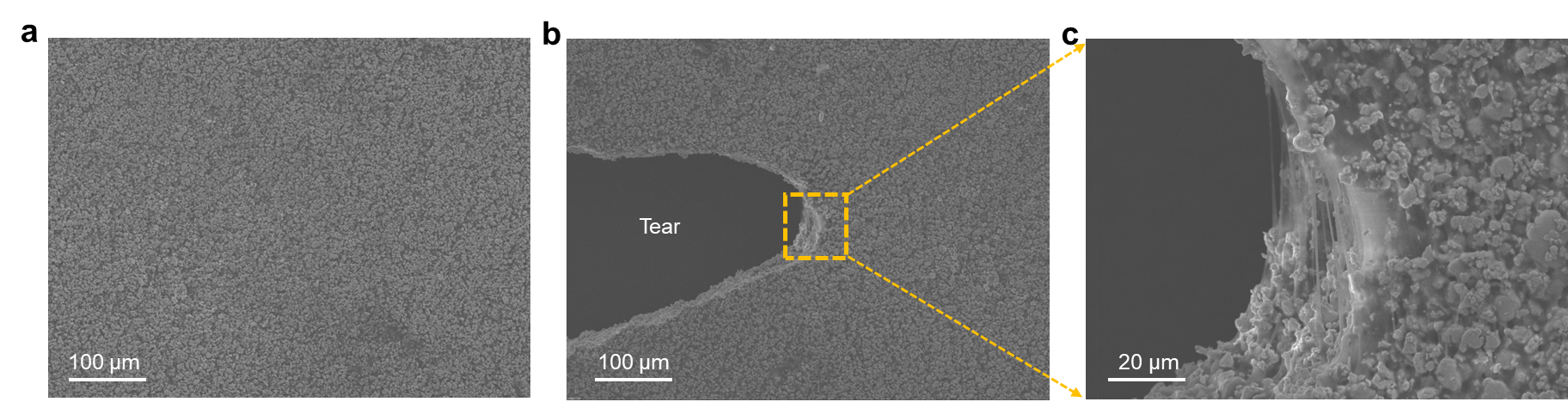


**Figure S17.** The SEM images of the AgFL-AgNS-SR_DDTP_7wt%_ nanocomposite (AgFL-AgNS = 40 vol%, THF peroxide = 0.045 M). **a** Before stretching. **b-c** After breakage at 57% strain.

**
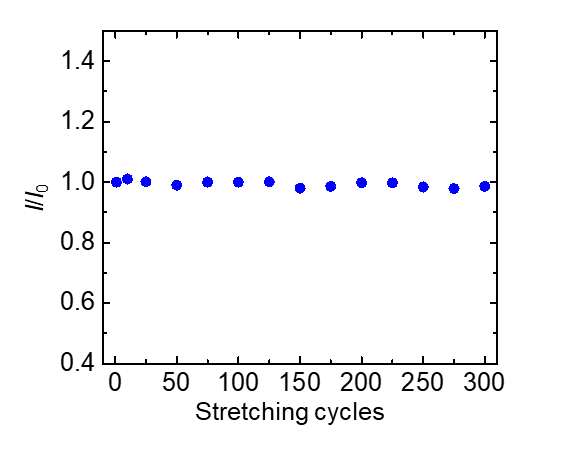
**

**Figure S18.** The normalized current of the AgFL-AgNS-SR_DDTP_7wt%_ nanocomposite (AgFL-AgNS = 40 vol%, THF peroxide = 0.045 M) at 40% strain is shown during 300 stretching cycles (maximum strain = 40%, crosshead speed = 5 mm/min, applied voltage = 10 mV).


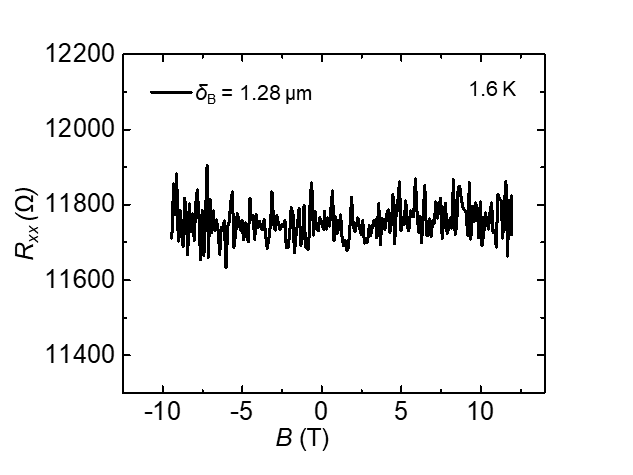


**Figure S19.** Magnetoresistance of the AgFL-SR_DDTP_7wt%_ nanocomposite (AgFL= 40 vol%). The barrier width is 1.28 μm.


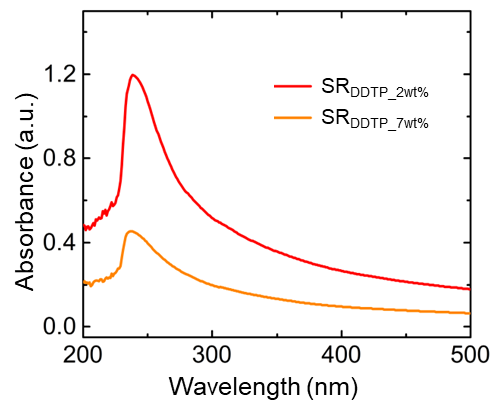


**Figure S20.** The absorption spectra of SR_DDTP_2wt%_ and SR_DDTP_7wt%_.


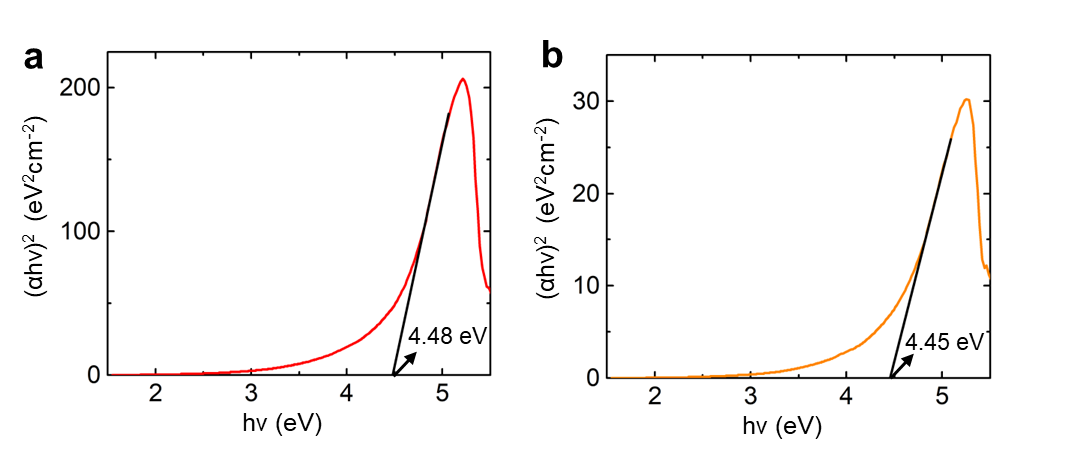


**Figure S21.** Tauc plot analysis. The optical bandgap is calculated by the linear fitting using the direct transition model, 𝛼ℎ𝜈 = (ℎ𝜈 − 𝐸_g_)^0.5^. The *α* is the absorption coefficient, *hν* is the photon energy, *A* is the constant, and *E*_g_ is the bandgap.^[6]^ **a** SR_DDTP_2wt%_. **b** SR_DDTP_7wt%_.

**Table S1.** The electrical conductivity (*σ*) at 0% strain and normalized resistance change (*ΔR/R*_0_) at 50% strain of the AgFL-AgNS-SR_DDTP_7wt%_ (AgFL-AgNS = 40 vol%, THF peroxide = 0.045 M) nanocomposite are compared with those of stretchable conductive nanocomposites in literature.

| **Filler** | **Matrix polymer** | ***σ***  **at 0% strain**  **(Scm^-1^)** | ***ΔR/R*_0_**  **at 50% strain**  **(%)** | **Reference** |
| --- | --- | --- | --- | --- |
| AgFL-AgNS | SR_DDTP_7wt%_ | 51,710 | 0 | This work |
| AgFL-AgNS | SR | 12 | 0* | [1] |
| Ag nanowire | poly(styrene-ethylene-butylene-styrene) | 120,000 | 300 | [7] |
| Ga-In liquid metal | Acrylic tape | 20,600 | ~1 | [8] |
| AgFL | Flourine rubber | 6168 | 200 | [9] |
| Ag-Au-Pt nanowires | Styrene-ethylene-butadiene-styrene | 11,000 | 100 | [10] |
| AgFL-GaIn | Ethylene vinyl acetate | 8331 | 10 | [11] |
| Polymerized eutectic GaIn | 2-hydroxyethyl acrylate | 2500 | ~1 | [12] |
| Ag nanowire-AgFL-Ag nanoparticle | poly(styrene-ethylene-butylene-styrene) | 31,000 | 0.4 | [13] |
| Au nanoparticle | Polyurethane | 15,700 | 100 | [14] |
| Graphene-AgFL | Polydimethylsiloxane | 1350 | 400 | [15] |
| AgFL-GaIn | Styrene-isoprene block copolymer | 7020 | 12 | [16] |
| Ag nanowire | poly(styrene-ethylene-butylene-styrene) | 122,120 | ~4 | [17] |

* The resistance change at 30% strain. The specimen was broken when the strain was greater than 30%.

**Supporting Note 1 Simmons approximation model analysis**

The Simmons approximation model predicts the tunneling current density (*J*) between two metal electrodes separated by a thin insulating film as a function of the barrier height (*λ*_B_) and barrier width (*δ*_B_), when the applied bias (*V*) is sufficiently low.^[1,18]^

$J= \frac{3\left( 2m_{e}\lambda_{B} \right)^{\frac{1}{2}}}{2\delta_{B}} \left( \frac{e}{h} \right)^{2} V\exp\left( -\frac{4\pi\delta_{B}}{h} \left( 2m_{e}\lambda_{B} \right)^{\frac{1}{2}} \right)$ (3)

where *m_e_* is the effective mass of electron (9.11×10^−31^ kg), *h* is the Planck’s constant, and *e* is the elementary charge.

The ratio of the tunneling current before (*I*_0_) and after (*I*) stretching is then described by Equation 4.

$\frac{I}{I_{0}}= \frac{A}{A_{0}} \frac{\delta_{B, before stretching}}{\delta_{B, after stretching}}\exp\left( -\frac{4\pi(\delta_{B, after stretching}- \delta_{B, before stretching})}{h} \left( 2m\lambda_{B} \right)^{\frac{1}{2}} \right)$ (4)

where *δ*_B,before stretching_ is the barrier width (i.e., interparticle distance) at 0% strain, and *δ*_B,after stretching_ is the barrier width after stretching. The cross-sectional area of the rectangular thin film before and after stretching are *A*_0_ and *A*, respectively.

The *I*/*I_0_* can then be obtained as a function of axial strain (*ε*) and Poisson’s ratio (*ν*) as shown in Equation 5. Note that a more detailed derivation is published elsewhere.^[1,18]^ For simplicity, the thickness of the thin film is assumed to be constant since it is difficult to experimentally measure during stretching.^[1,18]^

$\frac{I}{I_{0}}= \frac{(1-\nu\varepsilon)}{(1+\varepsilon)} \exp\left( -\frac{4\pi\times\varepsilon\delta_{B,\mathrm{be}fore stretching}}{h} \left( 2m_{e}\lambda_{B} \right)^{\frac{1}{2}} \right)$ (5)

The Poisson’s ratio (*ν* = 0.36) of the AgFL-AgNS-SR_DDTP_2wt%_ nanocomposite (AgFL-AgNS = 40 vol%, THF peroxide = 0.045 M) is experimentally measured. The $\delta_{B,before stretching}$ (4.1 nm) is obtained from HRTEM images. The *λ_B_* is then obtained by fitting the strain-dependent experimental data with Equation 5 as shown in Figure 4e. Note that Equation 5 becomes identical to the Ohms law when *λ_B_* is negligible (~0 eV).^[1,18]^

$\frac{I}{I_{0}}= \frac{(1-\nu\varepsilon)}{(1+\varepsilon)}$ (6)

**References**

[1] C. M. Ajmal, S. Cha, W. Kim, K. P. Faseela, H. Yang, S. Baik, *Sci. Adv.* **2022**, *8*, eabn3365.

[2] G. H. Jeffery, Bassett, J., Mendham, J., Denney, R. C, *Vogel’s Textbook of Quantitative Chemical Analysis.* Longman Scientific & Technical, Essex, UK **1989**.

[3] J. Schweitzer, S. Merad, G. Schrodj, F. Bally-Le Gall, L. Vonna, *J. Chem. Educ.* **2019**, *96*, 1472.

[4] P. J. Flory, J. Rehner, Jr., *J. Chem. Phys.* **1943**, *11*, 521.

[5] R. A. Orwoll, P. A. Arnold, in *Physical Properties of Polymers Handbook* (Ed: J. E. Mark), Springer, New York **2007**.

[6] P. Makuła, M. Pacia, W. Macyk, *J. Phys. Chem. Lett.* **2018**, *9*, 6814.

[7] D. Jung, C. Lim, H. J. Shim, Y. Kim, C. Park, J. Jung, S. I. Han, S.-H. Sunwoo, K. W. Cho, G. D. Cha, D. C. Kim, J. H. Koo, J. H. Kim, T. Hyeon, D.-H. Kim, *Science* **2021**, *373*, 1022.

[8] S. Liu, D. S. Shah, R. Kramer-Bottiglio, *Nat. Mater.* **2021**, *20*, 851.

[9] N. Matsuhisa, D. Inoue, P. Zalar, H. Jin, Y. Matsuba, A. Itoh, T. Yokota, D. Hashizume, T. Someya, *Nat. Mater.* **2017**, *16*, 834.

[10] S.-H. Sunwoo, S. I. Han, D. Jung, M. Kim, S. Nam, H. Lee, S. Choi, H. Kang, Y. S. Cho, D.-H. Yeom, M.-J. Cha, S. Lee, S.-P. Lee, T. Hyeon, D.-H. Kim, *ACS Nano* **2023**, *17*, 7550.

[11] J. Wang, G. Cai, S. Li, D. Gao, J. Xiong, P. S. Lee, *Adv. Mater.* **2018**, *30*, 1706157.

[12] C. J. Thrasher, Z. J. Farrell, N. J. Morris, C. L. Willey, C. E. Tabor, *Adv. Mater.* **2019**, *31*, 1903864.

[13] D. Jung, C. Lim, C. Park, Y. Kim, M. Kim, S. Lee, H. Lee, J. H. Kim, T. Hyeon, D.-H. Kim, *Adv. Mater.* **2022**, *34*, 2200980.

[14] M. Gu, W.-J. Song, J. Hong, S. Y. Kim, T. J. Shin, N. A. Kotov, S. Park, B.-S. Kim, *Sci. Adv.* **2019**, *5*, eaaw1879.

[15] L. Zhu, Y. Wang, D. Mei, W. Ding, C. Jiang, Y. Lu, ACS Appl. Mater. Interfaces **2020**, *12*, 31725.

[16] P. A. Lopes, D. F. Fernandes, A. F. Silva, D. G. Marques, A. T. de Almeida, C. Majidi, M. Tavakoli, ACS Appl. Mater. Interfaces **2021**, *13*, 14552.

[17] D. Jung, Y. Kim, H. Lee, S. Jung, C. Park, T. Hyeon, D.-H. Kim, *Adv. Mater.* **2023**, *35*, 2303458.

[18] J. G. Simmons, *J. Appl. Phys* **1963**, *34*, 1793.
